# Computational Modeling Reveals Key Contributions of KCNQ and hERG Currents to the Malleability of Uterine Action Potentials Underpinning Labor

**DOI:** 10.1371/journal.pone.0114034

**Published:** 2014-12-04

**Authors:** Wing-Chiu Tong, Rachel M. Tribe, Roger Smith, Michael J. Taggart

**Affiliations:** 1 Institute of Cellular Medicine, Newcastle University, Newcastle upon Tyne, United Kingdom; 2 Division of Women's Health, King's College London and King's Health Partners, London, United Kingdom; 3 Hunter Medical Research Institute, University of Newcastle, New Lambton, New South Wales, Australia; University of Tennessee Health Science Center, United States of America

## Abstract

The electrical excitability of uterine smooth muscle cells is a key determinant of the contraction of the organ during labor and is manifested by spontaneous, periodic action potentials (APs). Near the end of term, APs vary in shape and size reflecting an ability to change the frequency, duration and amplitude of uterine contractions. A recent mathematical model quantified several ionic features of the electrical excitability in uterine smooth muscle cells. It replicated many of the experimentally recorded uterine AP configurations but its limitations were evident when trying to simulate the long-duration bursting APs characteristic of labor. A computational parameter search suggested that delayed rectifying K^+^ currents could be a key model component requiring improvement to produce the longer-lasting bursting APs. Of the delayed rectifying K^+^ currents family it is of interest that KCNQ and hERG channels have been reported to be gestationally regulated in the uterus. These currents exhibit features similar to the broadly defined uterine *I*_K1_ of the original mathematical model. We thus formulated new quantitative descriptions for several *I*_KCNQ_ and *I*_hERG_. Incorporation of these currents into the uterine cell model enabled simulations of the long-lasting bursting APs. Moreover, we used this modified model to simulate the effects of different contributions of *I*_KCNQ_ and *I*_hERG_ on AP form. Our findings suggest that the alterations in expression of hERG and KCNQ channels can potentially provide a mechanism for fine tuning of AP forms that lends a malleability for changing between plateau-like and long-lasting bursting-type APs as uterine cells prepare for parturition.

## Introduction

The timely onset and maintenance of regular contractions of the uterus are necessary features for ensuring successful parturition and safe delivery of a baby and placenta. These contractions are driven by episodic, spontaneous myogenic action potentials (APs) that exhibit a broad spectrum of form and duration, the variability of which is likely to be beneficial in facilitating the co-ordination of uterine contractile effort during labor. For example, as pregnancy progresses uterine APs become more frequent and regular, the duration of the APs progressively lengthens [1] and they are often manifested as bursting APs that can last for many seconds to minutes [2], thus contributing to a greater time-averaged force of uterine contractions at parturition. Although more frequently shown in the recordings of multi-cellular uterine tissues, these APs have also been reported in single uterine smooth muscle (myometrial) cells isolated from near-labor, and in-labor, animals and humans [2], [3]. The quest for a deeper understanding of the mechanisms that regulate these changes in uterine AP forms would benefit from computational biology approaches. A recently established mathematical model for uterine smooth muscle cell (USMC) electrical excitability is a useful starting point for these considerations [4]. The electrogenic components of this model were largely based on published data from uterine and other smooth muscles. These components were validated by experimental data and the model reproduced several published types of AP forms (spike, plateau and short bursts of spikes) of variable duration ≈0.5 – 10 secs. However, despite showing these positive simulation performances, this model was limited in its ability to computationally reproduce all uterine AP behaviors, in particular, the very long duration (tens of sec to mins) bursting-type APs that have been experimentally noted in uterine smooth muscle cells from late pregnancy [1]–[3]. Also, another related problem is that the membrane voltage failed to repolarize upon cessation of the stimulation when it had remained at a depolarized level for too long, such as during attempts to simulate a prolonged AP. These limitations restrict the use of the cell model to simulating uterine AP responses of shorter duration.

Our aims in this study, therefore, were two-fold. First, to identify membrane currents that are essential for forming the characteristics of long duration bursting-type APs with full repolarization capacity that are, presently, lacking in the uterine cell model. Second, to perform simulation experiments to explore the possible mechanistic roles of these components in the malleability of uterine cell excitability.

## Results and Discussion

### Insight from one-at-a-time sensitivity measures

**Figure 1:**
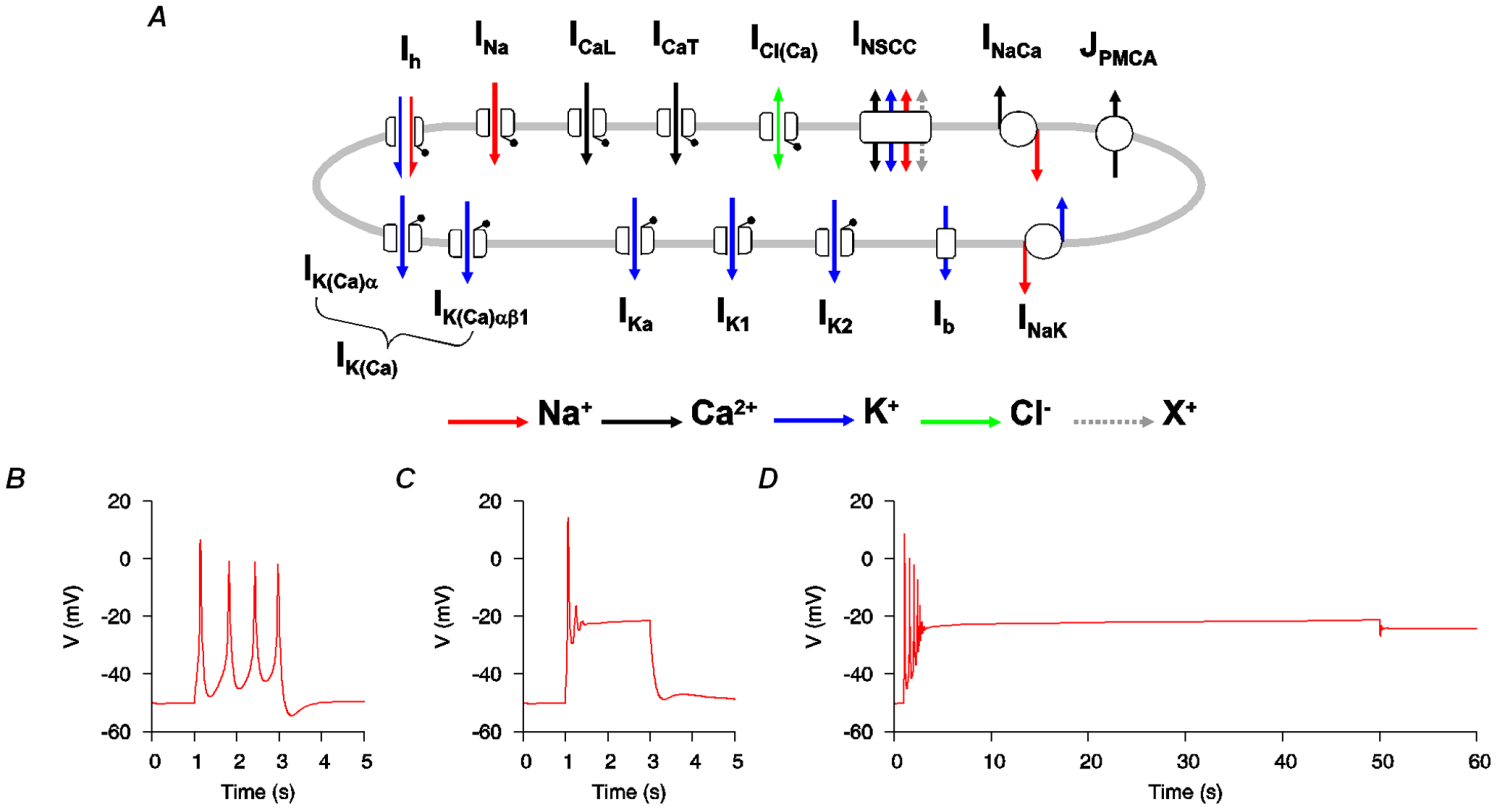
Electrogenic components of the initial USMC model. *A*, Schematic representation of the 14 ion channels/exchangers that contributed to the initial USMC model of myometrial electrical excitability [4]. *B-D*, this USMC model [4] reproduced different AP forms of short duration but it could not simulate long-lasting APs. *B*, short bursting-type AP evoked by a 2 s current clamp stimulus (*I*st) of -0.2 pA pF^−1^. *C*, short plateau-type AP evoked by a 2 s *I*st of −0.5 pA pF^−1^. *D*, an attempt to simulate a long duration AP with a 50 s *I*st of −0.25 pA pF^−1^ resulted in a plateau-like AP that failed to repolarize at the end of the stimulus. All three simulated APs were initiated from the same initial conditions.

The original USMC model [4] (the electrogenic components are depicted in Figure 1A) reliably simulated uterine bursting-type and plateau-type APs of short duration, examples of which are given in Figure 1B-C. However, to computationally reproducing APs of long duration bursting was a challenge (Figure 1D). We, therefore, performed a simple computational parameter sensitivity analysis to assess which kinetic properties would have the ability to improve the functionality of the model.

**Figure 2:**
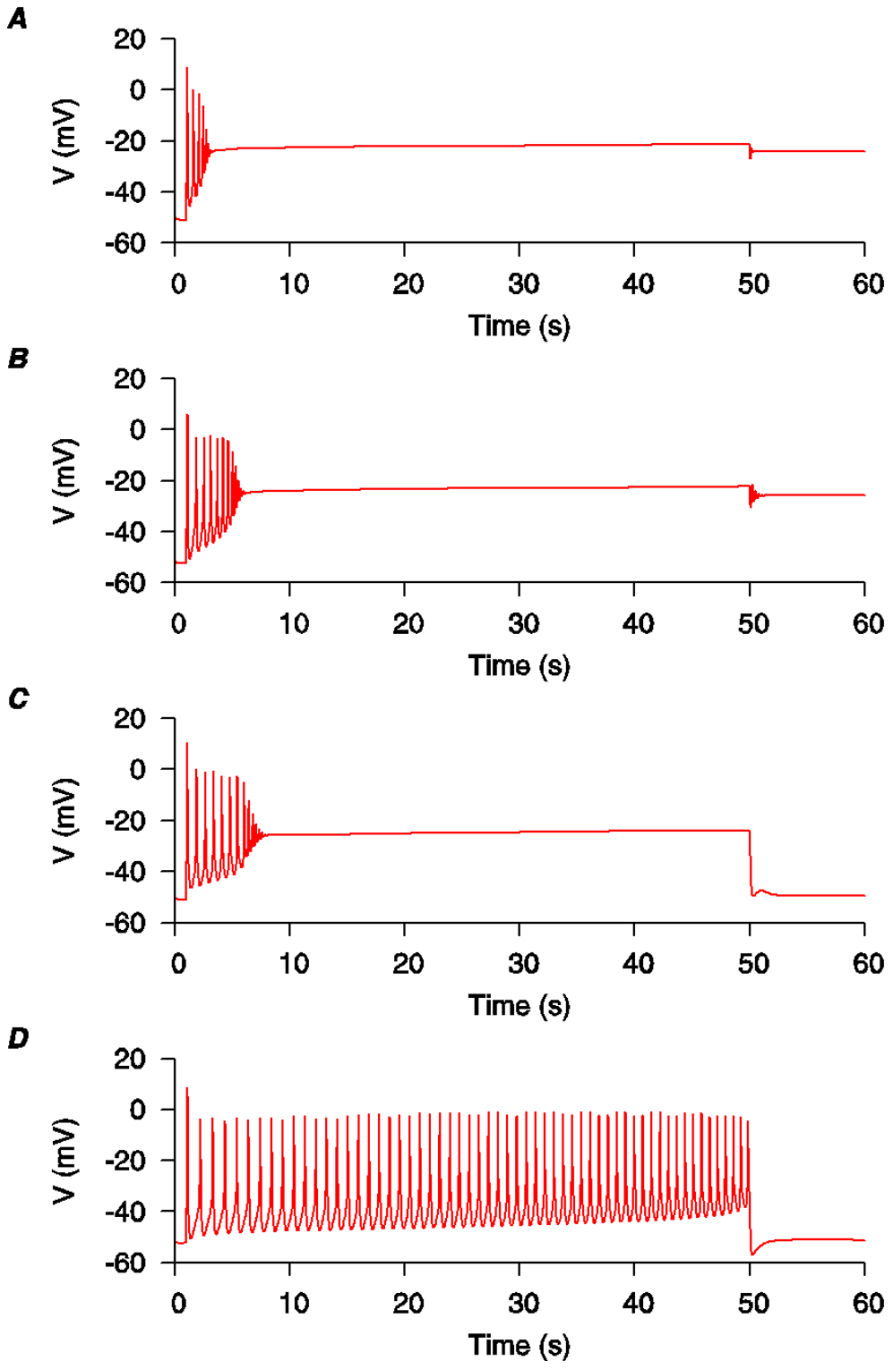
Altering the kinetic properties of *I*_K1_ in the initial USMC model was found to favor longer bursting-type APs. *A*, the standard configuration of the initial USMC model [4] as depicted in Figure 1D. *B*, increasing the *I*_K1_ conductance (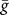
_K1_ from 0.52 nS pF^−1^ to 0.64 nS pF^−1^) prolonged the initial bursting phase a little without enabling repolarization of the AP. *C*, alternatively, scaling the *I*_K1_ time constants (activation: *τ_q_*, 2X; inactivation: *τ_r_*_1_, 20X and *τ_r_*_2_, 20X) also prolonged the bursting phase and facilitated the membrane voltage repolarization at the end of the stimulus. *D*, by changing both the conductance and time constants of *I*_K1_ together, the bursting persisted until the end of the stimulus and the membrane voltage repolarized on stimulus cessation.

Parameters such as the maximal conductances (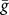), the half activations and inactivations (*V*_0.5_), the slope factors (*k*) of the steady-state activations and inactivations, and the time constant functions of activation or inactivation (*τ*), were changed one-at-a-time while holding the others at their fixed values. At each alteration, the influence of the parameter change on the model output was assessed by examining the form and duration of the initial burstings in a simulated AP evoked with a 50 s current clamp stimulus. The results were ranked and evaluated. We found that no single intrinsic parameter alteration of the ionic currents could produce a transition from short to long duration bursting APs. However, modification of some parameters could prolong the initial spiking period during an AP; in particular, changes in the maximal conductance (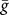
_K1_) and the fast inactivation time constant (*τ*_r1_) of the uterine delayed rectifying K^+^ current, *I*_K1_ (not to be confused with the time-independent inward rectifying potassium current commonly designated also as *I*_K1_ in cardiac cells). Figure 2 shows the result of changing the kinetic properties of *I*_K1_ on the uterine AP. Compared to the control case (Figure 2A), the initial bursting duration was slightly increased when 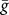
_K1_ was increased (Figure 2B). A similar result was obtained when *τ*_r1_ was scaled up but there were no apparent changes when either the activation time constant (*τ*_q_) or the slow inactivation time constant (*τ*_r2_) of *I*_K1_ was changed. Since all these parameters influence the same ionic current, *I*_K1_, their combined influences could exert more noticeable changes on the AP form than changing one parameter in isolation. Thus, we examined the effect of *I*_K1_ having overall slower dynamics (*τ*_q_, 2X; *τ*_r1_, 20X; *τ*_r2_, 20X) but this only slightly prolonged the initial bursting duration (Figure 2C). However, when an overall slower *I*_K1_ was combined with a larger conductance then the bursting continued until the end of the stimulus (Figure 2D). The resultant AP resembled the experimentally recorded long bursting behaviors [1], [2]. This simple parameter analysis provided an insight that, in order to simulate the long-lasting bursting APs, the uterine cell model required larger and slower delayed rectifying K^+^ currents. To make such changes we needed to either describe a known mechanism for causing such changes in uterine *I*_K1_ or replace the uterine *I*_K1_ with other molecularly identified K^+^ currents with such biophysical properties. There is no experimental information to support the former, and so we proceeded with the latter.

Uterine whole-cell voltage-gated K^+^ currents have only been roughly categorized based on their inactivation properties and their sensitivity to different broad-spectrum potassium channel blockers. For example, the uterine *I*_K1_ has been described as a prominent voltage-gated, delayed rectifying K^+^ current rather loosely identified by its pharmacological profile and activation/inactivation dynamics [5]. Under voltage-clamp conditions, the activation threshold where *I*_K1_ first appears is between −60 mV to −40 mV, it has a half activation of ≈1.2 mV and a half-inactivation of ≈−65 mV. The dynamics of *I*_K1_ are slow and the decay of the current can take as much as 10 secs. Also, *I*_K1_ is sensitive to 10 mM TEA but insensitive to 5 mM 4-AP. Given these rather broad characterizations, there are contributions to this current that are likely to come from a number of molecularly distinct channels. When considering the possible identity of these channels we noted the following:

(i) In rat uteri at late pregnancy mRNA encoding a very slowly activating K^+^ channel increased in expression. This K^+^ current shares similarities to the cardiac delayed rectifying currents *I*_Ks_ and *I*_Kr_ [6], [7].

(ii) The delayed rectifying cardiac *I*_Ks_ consists of the KCNQ voltage-gated pore-forming channel(s) and KCNE subunits and recent evidence suggests that uterine transcripts encoding KCNQ and KCNE subunits are gestationally regulated [8].

(iii) KCNQ activation time constants and conductances are subject to modulation by the pattern of KCNE subunit co-expression [9].

(iv) KCNQ potassium channels can be blocked by XE991 and XE991-sensitive currents in portal venous smooth muscle showed broadly similar characteristics as uterine smooth muscle *I*_K1_ with a half-activation of 7 mV and a half-inactivation of −54 mV; the activation threshold is −30 mV [10].

(v) KCNQ currents have pharmacological profiles similar to *I*_K1_ as many members of the KCNQ channels are sensitive to TEA and insensitive to 4-AP [11], [12].

(vi) A major contributor to the delayed rectifying cardiac *I*_Kr_ is the human *Ether-à-Go-Go*-related (hERG) channel current. Recent evidence has shown that hERG channel subunits are expressed in uterine cells and the currents sensitive to hERG inhibitors, e.g. E4031 or dofetilide, are functionally suppressed at late pregnancy [13], [14].

(vii) The dynamics of hERG currents are comparable to *I*_K1_: the activation threshold is −50 mV [15], [16]; a half-inactivation is between -94 mV and −48 mV [15]–[18] and, depending on the recording conditions, the half-activation of hERG varies between −31 mV to 17 mV [15]–[21].

These data, together with the results of the parameter analysis, led us to speculate that KCNQ and hERG currents may form part of the uterine *I*_K1_ current. Our next task was therefore to assess how best to incorporate this experimental information into the USMC model. However, there are no experimental measurements specific to uterine smooth muscle cells on the dynamics of KCNQ currents and there is only limited information about hERG-like currents in these cells. Thus, we next sought to (i) establish quantitative descriptions of the biophysical details from clonal data for KCNQ and hERG currents and (ii) modify the USMC model by substituting *I*_K1_ with KCNQ and hERG currents.

### Formulation of mathematical descriptions of KCNQ1, KCNQ4, KCNQ5 and hERG currents

The KCNQ subunits form the pore of K^+^ channels. There are five members in the KCNQ family: Q1–Q5. In mouse uterine tissues, the mRNA expressions of all KCNQ subunits were suppressed from early to mid-gestation until late term when KCNQ1, KCNQ4 and KCNQ5 mRNA levels were increased [8]. Based on this information, we decided to formulate descriptions for currents determined by KCNQ1 (*I*_KCNQ1_), KCNQ4 (*I*_KCNQ4_) and KCNQ5 (*I*_KCNQ5_).

There are at least three main variants of hERG protein (hERG1-3), the expressions of which impose different kinetic characteristics on the channel current. The full-length hERG (also known as hERGA or hERG1a) is found in all types of muscles cells. hERG1a is known to form a N-terminus splice variant (known as hERGb or hERG1b), which shows faster activation and deactivation, and smaller tail currents compared to hERG1a [22]. A C-terminus splice variant of hERG (also known as hERGC [19], HERG_USO_ [20] or erg-sm [16]) has also been reported, including from smooth muscle. However, the descriptions of its characteristics vary from being non-functional channels to fully conducting channels. Greenwood et al. (2009) [13] showed that both hERG1a and hERG1b, but not hERG2 or hERG3, were expressed in mouse myometrium with hERG1a being the most abundant isoform. Therefore, we developed formulations for hERG current (*I*_hERG_) based upon published datasets for hERG1a.

Detailed information on the kinetics and steady-state voltage (*V*)-dependent parameters of *I*_KCNQ_ or *I*_hERG_ has been obtained from clonal data from heterologous expression cell systems for each channel subunit: KCNQ1 [10], [23]–[35], KCNQ4 [25], [30]–[32], [34], [36]–[39], KCNQ5 [31], [32], [34],[36],[38]–[42] and hERG [15], [16], [19], [20], [43]. Therefore, mathematical descriptions of the channel biophysical details were developed from these data. Mathematical descriptions of the biophysical characteristics of these currents are given in Appendix S1: for *I*_KCNQ1_ see equations 1–13; for *I*_KCNQ4_ see equations 14–20; for *I*_KCNQ5_ see equations 21–32; for *I*_hERG_ see equations 33–41.

**Figure 3:**
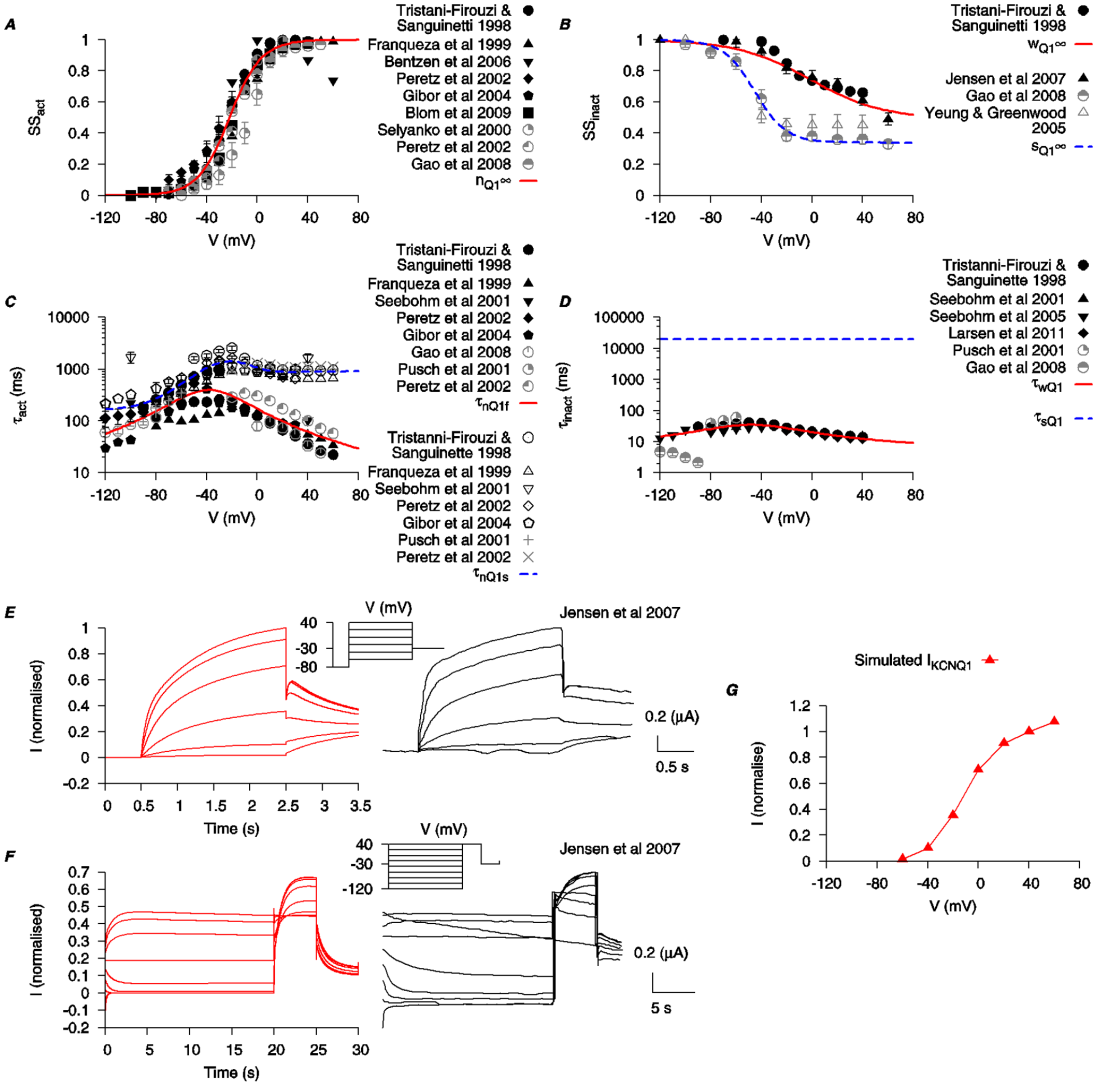
Biophysical characteristics of KCNQ1 current. Properties of *I*_KCNQ1_ were developed using experimental data from human KCNQ1 clones expressed in *Xenopus laevis* oocytes (*black*) and Chinese hamster cell (*gray*) [23]–[35], as well as KCNQ-like currents in mouse smooth muscle cells [10]. *A*, *V*-dependent activation steady state (*n*_Q1∞_). *B*, *V*-dependent steady states for fast inactivation (*w*_Q1∞_) and slow inactivation (*s*_Q1∞_). *C*, *V*-dependent activation time constants (*τ_n_*_Q1f_ and *τ_n_*_Q1s_). *D*, fast (*τ_w_*_Q1_) and slow (*τ_s_*_Q1_) inactivation time constants. *τ_w_*_Q1_ is *V*-dependent and fitted to experimental data [23], [26], [27], [30], [33], [35]. *τ_s_*_Q1_ is set as a constant at 50 s. This value is chosen to best reproduce the published time tracings from Jensen et al. (2007) [32]. *E* and *F*, simulated time tracings of *I*_KCNQ1_ with two different voltage-clamp protocols from [32] (*insets*) and the experimental time tracings are shown for comparison. (Experimental tracing adapted with permission from Jensen et al. (2007); copyright 2007, Biophysical Society.) *G*, simulated *I-V* relationship of *I*_KCNQ1_ using the same voltage-clamp protocol as in *E*. All *I-V* data are normalized to the maximal current value at *V* = 40 mV.

The equations for *I*_KCNQ1_ incorporate two activation gating variables (*n*_Q1f_ and *n*_Q1s_) and two inactivation gating variables (*w*_Q1_ and *s*_Q1_). Steady-state values for activation and inactivation are shown in Figure 3A-B. The time constants of activation (*τ_n_*_Q1f_ and *τ_n_*_Q1s_) and inactivation (*τ_w_*_Q1_ and *τ_s_*_Q1_) are depicted in Figure 3C-D and were each obtained by fitting the raw data current tracings from the literature [23]–[35]. *τ_w_*_Q1_ is *V*-dependent and fitted to experimental data [23], [26], [27], [30], [33], [35]. *τ_s_*_Q1_ is set as a constant at 50 s. This value was chosen to best reproduce the published time tracings from Jensen et al. (2007) [32]. Simulated traces of *I*_KCNQ1_ under voltage-clamp conditions are presented in Figure 3E–F and show dynamic profiles similar to the raw data [32]. The simulated I–V relationship of *I*_KCNQ1_ is shown in Figure 3G.

**Figure 4:**
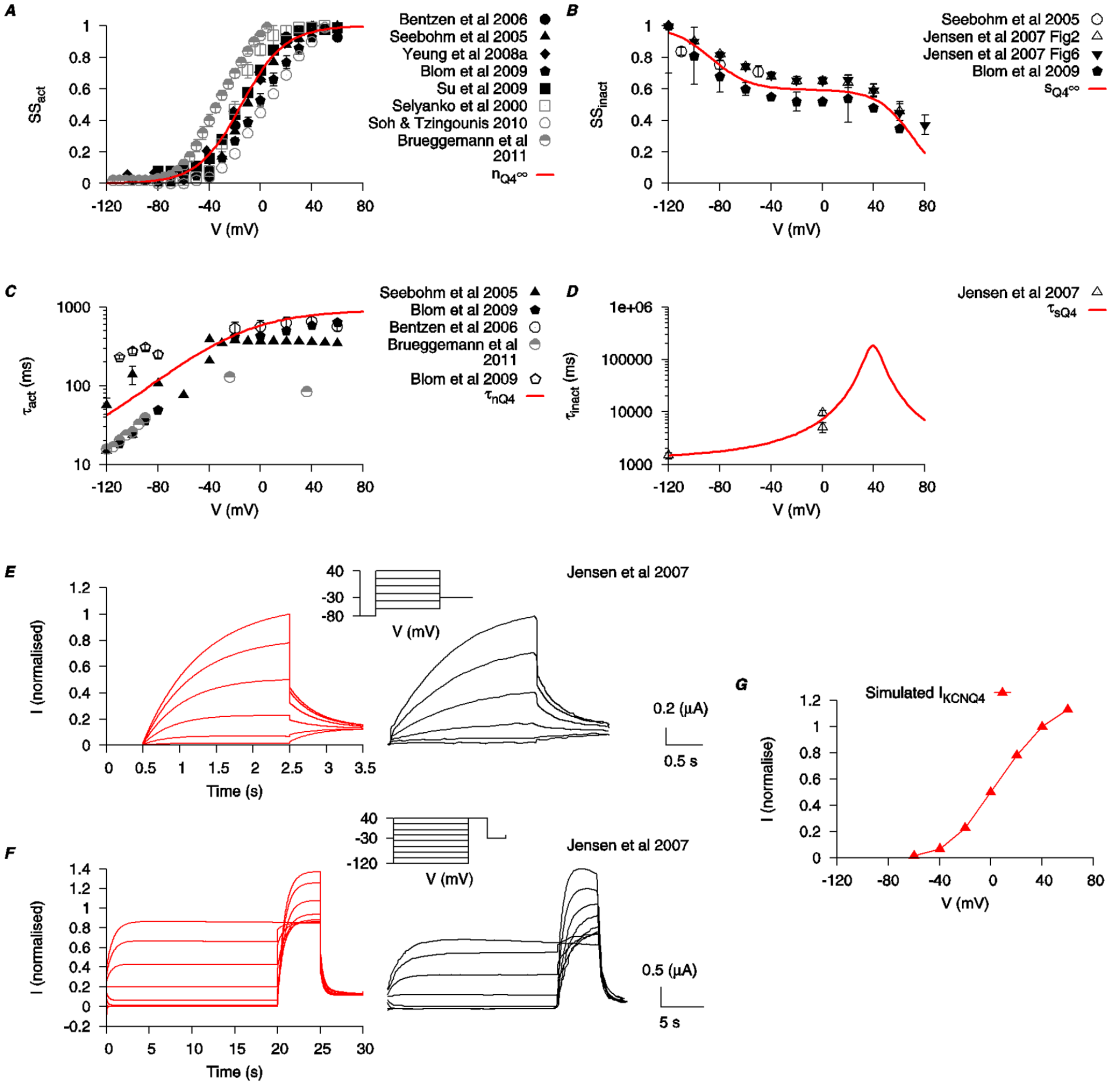
Biophysical characteristics of KCNQ4 current. Properties of *I*_KCNQ4_ are developed using experimental data from mammalian KCNQ4 clones expressed in *Xenopus laevis* oocytes (*black*) and other expression systems (*gray*) [25], [30]–[32], [34], [36]–[39]. *A*, *V*-dependent activation steady state (*n*_Q4∞)._ *B*, *V*-dependent inactivation steady state (*s*_Q4∞_). *C*, *V*-dependent activation time constant (*τ_n_*_Q4_). *D*, *V*-dependent inactivation time constant (*τ_s_*_Q4_). *E* and *F*, simulated time tracings of *I*_KCNQ4_ with two different voltage-clamp protocols from Jensen et al. (2007) [32] (*insets*) and the experimental time tracings are shown for comparison. (Experimental tracing adapted with permission from Jensen et al. (2007); copyright 2007, Biophysical Society.) *G*, simulated *I-V* relationship of *I*_KCNQ4_ using the same voltage-clamp protocol as in *E*. All *I-V* data are normalized to the maximal current value at *V* = 40 mV.

The equations for *I*_KCNQ4_ incorporate an activation (*n*_Q4_) and an inactivation (*s*_Q4_) gating variable. Steady-state values for activation and inactivation are shown in Figure 4A-B. Blom et al. (2009) [34] reported that KCNQ4 currents were best described with a single time constant for its activation within the voltage range of −40 mV to +40 mV, and two time constants for its deactivation. Thus we used a single time constant for activation (*τ_n_*_Q4_) and its voltage-dependency is depicted in Figure 4C. We modeled the current with one inactivation time constant (*τ_s_*_Q4_) (Figure 4D). Raw data concerning the dynamics of inactivation of *I*_KCNQ4_ are limited and therefore, for simplicity, a function was chosen to fit with the data available and which best reproduced the published raw data time tracings from Jensen et al. (2007) [32]. Simulations of raw data traces of *I*_KCNQ4_ under voltage-clamp conditions matched well to the experimental data [32] (Figure 4E–F) and the simulated I–V relationship is shown in Figure 4G.

**Figure 5:**
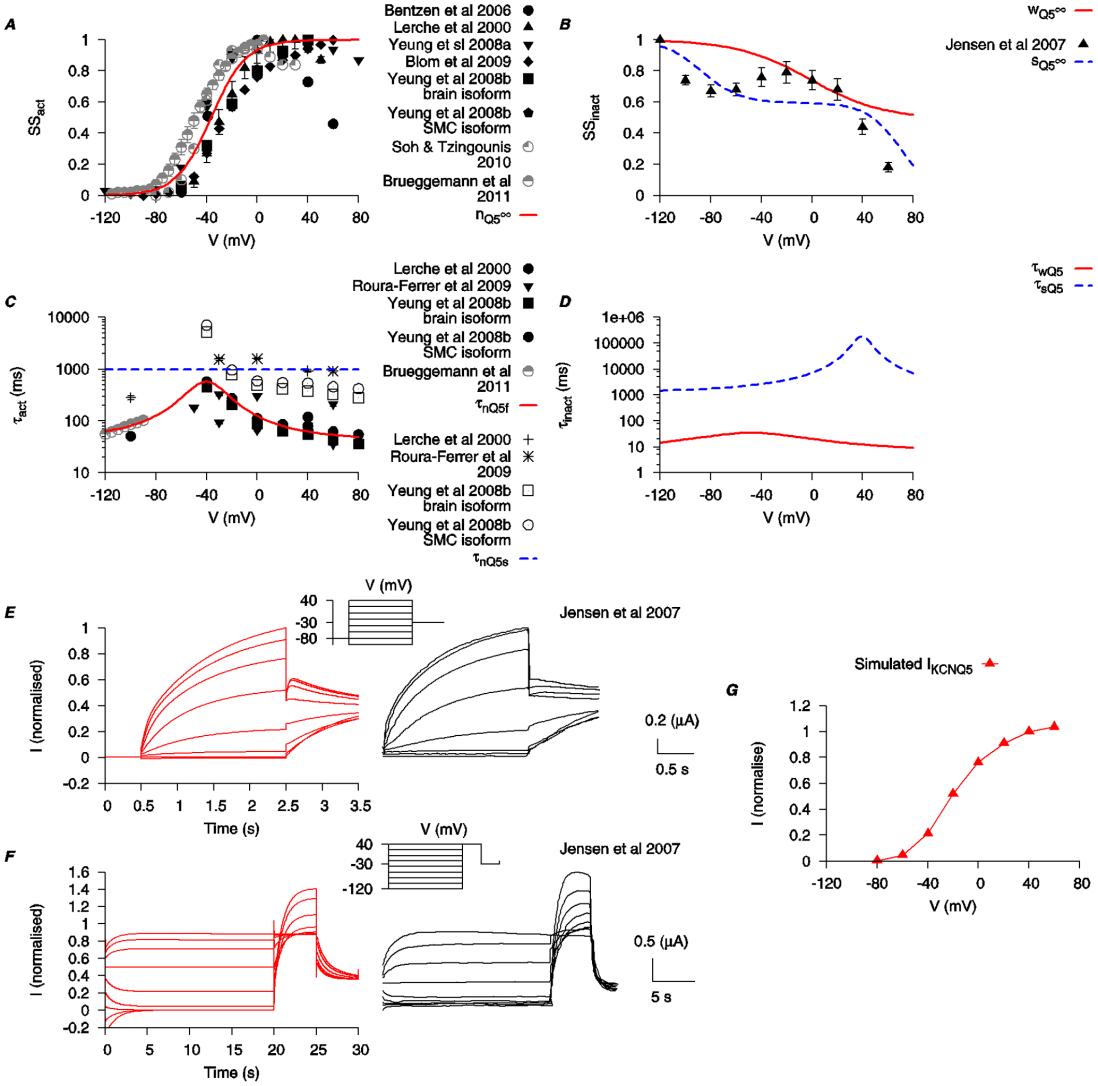
Biophysical characteristics of KCNQ5 current. Properties of *I*_KCNQ5_ are developed using experimental data from cloned mammalian KCNQ5 expressed in *Xenopus laevis* oocytes (*black*) and other expression systems (*gray*) [31], [32], [34], [36], [38]–[42]. *A*, *V*-dependent activation steady state (*n*_Q5∞_). *B*, *V*-dependent steady states for fast inactivation (*w*_Q5∞_) and slow inactivation (*s*_Q5∞_). *C*, *V*-dependent activation time constants (*τ_n_*_Q5f_ and *τ_n_*_Q5s_). *τ_n_*_Q5f_ is *V* -dependent but *τ_n_*_Q5s_ is set as a constant at 1 s. *D*, *V*-dependent time constants for fast inactivation (*τ_w_*_Q5_) and slow inactivation (*τ_s_*_Q5_). *E* and *F*, simulated time tracings of *I*_KCNQ5_ with two different voltage-clamp protocols (*insets*) used in Jensen et al. (2007) [32] and the experimental time tracings are shown for comparison. (Experimental tracing adapted with permission from Jensen et al. (2007); copyright 2007, Biophysical Society.) *G*, simulated *I*–*V* relationship of *I*_KCNQ5_ using the same voltage-clamp protocol as in *E*. All *I*–*V* data are normalized to the maximal current value at *V* = 40 mV.

The equations for *I*_KCNQ5_ incorporate two activation gating variables (*n*_Q5f_ and *n*_Q5s_). Steady-state values for the activation are shown in Figure 5A. The dynamics of the activation are described by two time constants (*τ_n_*_Q5f_ and *τ_n_*_Q5_s, Figure 5C). However, it was less straightforward to mathematically describe the steady-state *V*-dependent inactivation of *I*_KCNQ5_. For example, using functions that fitted well with the reported steady-state inactivation data of Jensen et al. (2007) [32], it was not possible to replicate the raw data current tracings from the same publication. Moreover, the hooked tail currents of *I*_KCNQ5_ shown in Jensen et al. (2007) [32] suggested that, in order to reproduce this feature, an inactivation faster than the activation may be required. Without sufficient other information concerning the inactivation dynamics of *I*_KCNQ5_, we have, therefore, used the fast inactivation of *I*_KCNQ1_ and the slow inactivation of *I*_KCNQ4_ to represent the fast and slow inactivation conditions for *I*_KCNQ5_, *i.e.*, *w*_Q5∞_ =  *w*_Q1∞_, *τw*_Q5_ =  *τw*_Q1_, *s*_Q5∞_ =  *s*_Q4∞_ and *τ_s_*_Q5_ = *τ_s_*_Q4_. With these adjustments (Figure 5B,D), the simulated *I*_KCNQ5_ profiles were satisfactory compared to the published experimental raw data time tracings (Figure 5E–F). The simulated I–V relationship is shown in Figure 5G.

**Figure 6:**
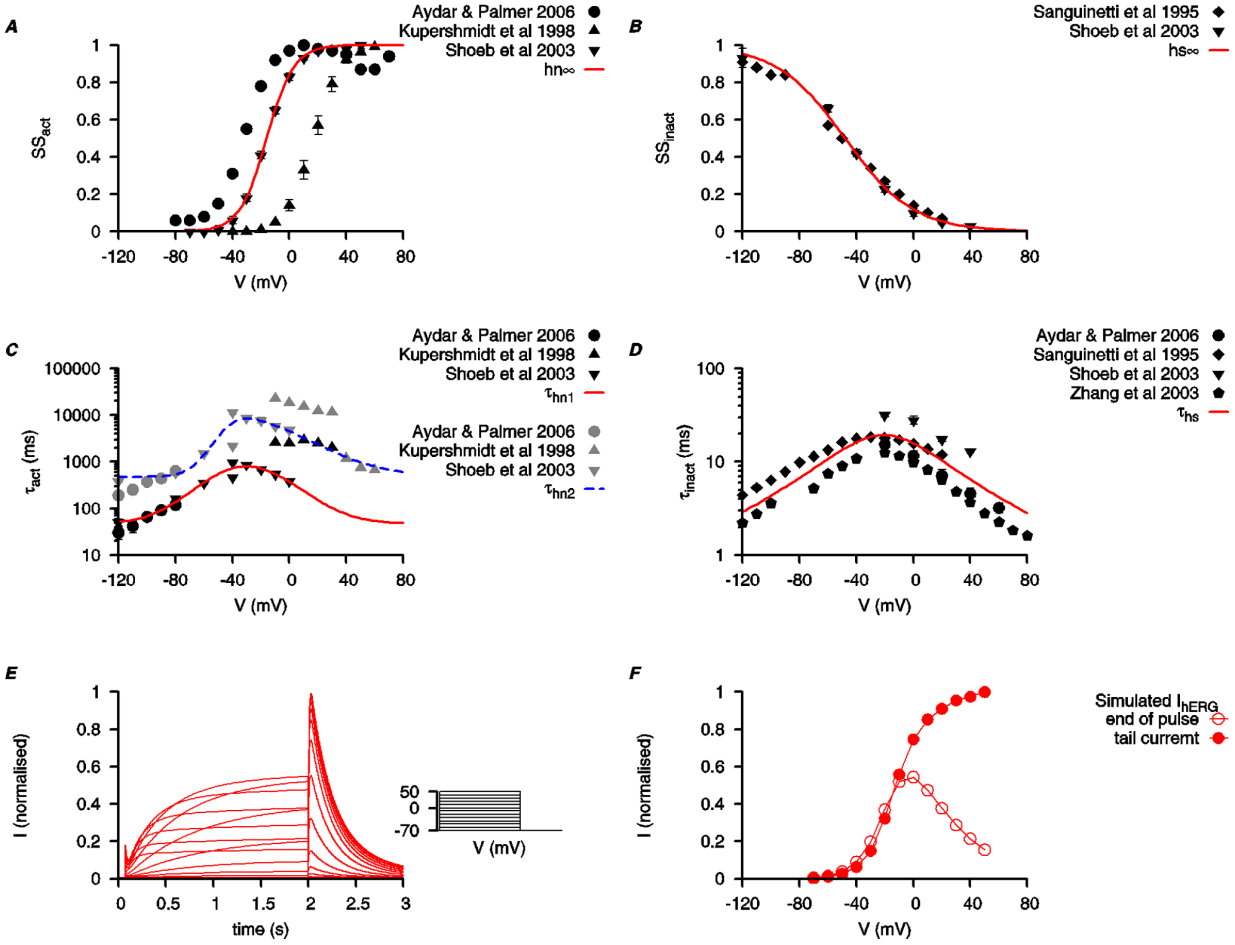
Biophysical characteristics of hERG current. Properties of *I*_hERG_ for the myometrial cell model are developed using experimental data from full length hERG clones expressed in different expression systems [15], [16], [19], [20], [43]. *A*, *V*-dependent activation steady state (*h_n_*_∞_). *B*, *V* -dependent inactivation steady state (*h_s_*_∞_). *C*, *V*-dependent activation time constants (*τ_hn1_* and *τ_hn2_*). *D*, *V*-dependent inactivation time constant (*τ_hs_*). E, simulated time tracings of *I*_hERG_. Currents were evoked from a holding potential (*V*_h_) of −70 mV to various voltage steps (*V*_step_) for 2 s then stepped back to *V_h_*. *F*, simulated *I-V* relationships of *I*_hERG_ using the same voltage-clamp protocol as in E. Both the currents at the end of *V*_step_ (*empty points*) and the following maximal tail currents after the end of *V*_step_ (*solid points*) were plotted against *V*_step_. In both E and F, all data are normalized to the peak tail current value at *V* = 60 mV.

The equations for hERG incorporate two activation gating variables (*h_n1_* and *h_n2_*) and an inactivation gating variable (*h_s_*). Steady-state values for activation and inactivation are shown in Figure 6A–B. The time constants of activation (*τ_hn1_* and *τ_hn2_*) and inactivation (*τ_hs_*) are illustrated in Figure 6C–D. The kinetics of hERG currents have rather slow activation and deactivation (msec to sec) profiles but rapid inactivation (msec). As a result, the time tracings of raw data of *I*_hERG_ under voltage-clamp conditions often show large ‘hook’ tail currents when the command voltage is stepped down from a depolarized level. Simulations of these current tracings are shown in Figure 6E. The corresponding I–V relationships, of either the end-of-pulse current or the peak tail current, are depicted in Figure 6F.

### Substitution of I_K1_ in the USMC model by individual KCNQ or hERG currents

As rationalized above, in points (i)–(vii), *I*_KCNQ_ or *I*_hERG_ or both could be constituent components of the global, rather ill-defined uterine K^+^ current measured as *I*_K1_. We proceeded, therefore, to assess if *I*_K1_ in the USMC model could be replaced by *I*_KCNQ_ or *I*_hERG_ or a combination of each. As there is little information about the current densities for *I*_KCNQ_ or *I*_hERG_ in uterine cells, and the expression of uterine KCNQ and hERG subunits changes during pregnancy, we fitted the values of their maximal conductances so that the simulated maximal whole cell K^+^ currents matched the experimental value from Wang et al. (1998) [44].

First, we attempted to completely replace *I*_K1_ in the USMC model with, in turn, *I*_KCNQ1_, *I*_KCNQ4_, *I*_KCNQ5_ or *I*_hERG_. In particular, we were interested in seeing if this could change the AP forms. Each of these maneuvers resulted in the simulation of interesting long-duration AP forms, including a long bursting-type AP form (Figure S1). However, in each case, the corresponding simulations of whole cell K^+^ current tracings and I–V plots did not match the experimental data (middle and right panels of Figure S1B-E). In native USMC cells [5], [44], and in the original USMC model [4], the majority of myometrial *I*_K1_ would be inactivated by a holding potential (*V*_h_) of −40 mV due to the negative half inactivation of *I*_K1_. So, under voltage-clamped conditions, a larger outward K^+^ current would be evoked from a *V*_h_ of −80 mV compared to the current evoked from a *V*_h_ of −40 mV (Figure S1A middle panel). However, with complete replacement of *I*_K1_ by individual *I*_KCNQ1_, *I*_KCNQ4_, *I*_KCNQ5_ or *I*_hERG_, the resultant whole cell steady-state K^+^ current amplitude was the same when evoked from either *V*_h_. This was a deviation from the previously matched experimental results. Nonetheless, this exercise showed that *I*_KCNQ_ or *I*_hERG,_ could contribute to the development of long duration burstings APs in uterine cells.

Next, we partially replaced *I*_K1_ in the cell model with, in turn, *I*_KCNQ1_, *I*_KCNQ4_, *I*_KCNQ5_ or *I*_hERG_, while ensuring the simulated whole cell K^+^ current matched to the experimental data from Wang et al. (1998) [44]. With the standard model configuration, before any changes were implemented, 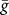
_K1_ =  0.52 nS pF^−1^ (Figure S2A). We proceeded with the partial substitution in steps. The level of *I*_K1_ was first reduced by changing 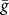
_K1_ to 0.24 nS pF^−1^ (Figure S2B). At this reduced level of *I*_K1_, the long-duration AP configuration shows only a plateau-type form with no initial bursting. However, the voltage-clamp experimental results for the whole cell K^+^ current from different holding potentials can still be reproduced and so this level of *I*_K1_ was chosen as a reasonable modification with which to investigate the effects of partially replacing *I*_K1_ by *I*_KCNQ_ or *I*_hERG_. Within the constrain of matching the experimental whole cell K^+^ currents (middle panels) and the *I-V* relationships (right panels) from Wang et al. (1998) [44], we identified the conductance ranges for *I*_KCNQ_ or *I*_hERG_ that favor long-duration bursting APs. With reduced *I*_K1_ and incremental additions of *I*_KCNQ1_, within a 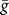
_KCNQ1_ range of 0.026 – 0.042 nS pF^−1^, complex AP forms could be simulated whereby the AP plateau was interspersed with periodic bursting (Figure S2B-E). Alternatively, with reduced *I*_K1_ and incremental additions of *I*_KCNQ4_, within a 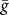
_KCNQ4_ range of 0.03216 − 0.04712 nS pF^−1^ there was an increasing tendency towards bursting behavior persisting throughout the AP albeit with diminishing amplitude (Figure S3C–E). With reduced *I*_K1_ and incremental additions of *I*_hERG_, within a 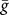
_hERG_ range of 0.112 – 0.208 nS pF^−1^, there was an increasing tendency towards improving the bursting behavior of long-duration AP (Figure S5C-E). In contrast, if the reduced *I*_K1_ was replaced with incremental additions of *I*_KCNQ5_, there was no observable change in the AP form from that of a plateau-type AP (Figure S4C-E, left panel).

The tendency of *I*_KCNQ1_, *I*_KCNQ4_ or *I*_hERG_, to improve the bursting potential of the AP form, suggested that each current individually - *I*_KCNQ1_, *I*_KCNQ4_ or *I*_hERG_ - could improve the capabilities of the USMC model in the manner we set out to achieve. For *I*_KCNQ5_ the situation appeared different to the other three currents in that after its partial replacement of *I*_K1_ the AP forms remained tonic and plateau-like (Figure S4). The effect of incremental inclusion of *I*_KCNQ5_ was to reduce the plateau potential and the resting membrane potential. Thus *I*_KCNQ5_ does not seem to play a role in bursting APs generation. In addition, there may be species-dependent differences in KCNQ5 expression patterns close to term and/or during labor with human myometrium expressing less KCNQ5 than KCNQ1 or KCNQ4 [8]. This, coupled to the rather negative membrane potentials for activation of the current (Figure 5A), led us to speculate that if *I*_KCNQ5_ is to have a role in USMC, it may be for setting the level of resting membrane potential or plateau potential or both rather than contributing to the macroscopic action potential generated K^+^ current. In our formulation of the original USMC model [4], a background K^+^ current, *I*_b_, was included to represent a collection of minor K^+^ currents with unknown kinetics that were necessary to hold the membrane potential at physiologically relevant resting levels. The above information suggested that the biophysically detailed *I*_KCNQ5_ may ably replace the rather vaguely defined *I*_b_. Indeed, when this was done, the cell model was still capable of reproducing all the previous validation results (data not shown). Moreover, with a small increase in *I*_KCNQ5_, the model repolarized at the end of the stimulation, while in the original cell model, a small increase of *I*_b_ did not cause the same behavior (data not shown). Furthermore, replacing *I*_b_ with *I*_KCNQ5_ did not affect the results of partially substituting *I*_K1_ by *I*_KCNQ1_, *I*_KCNQ4_ or *I*_hERG_ (data not shown). Thus, our first confirmed modification of the initial USMC cell model [4] was to replace *I*_b_ with *I*_KCNQ5_.

**Figure 7:**
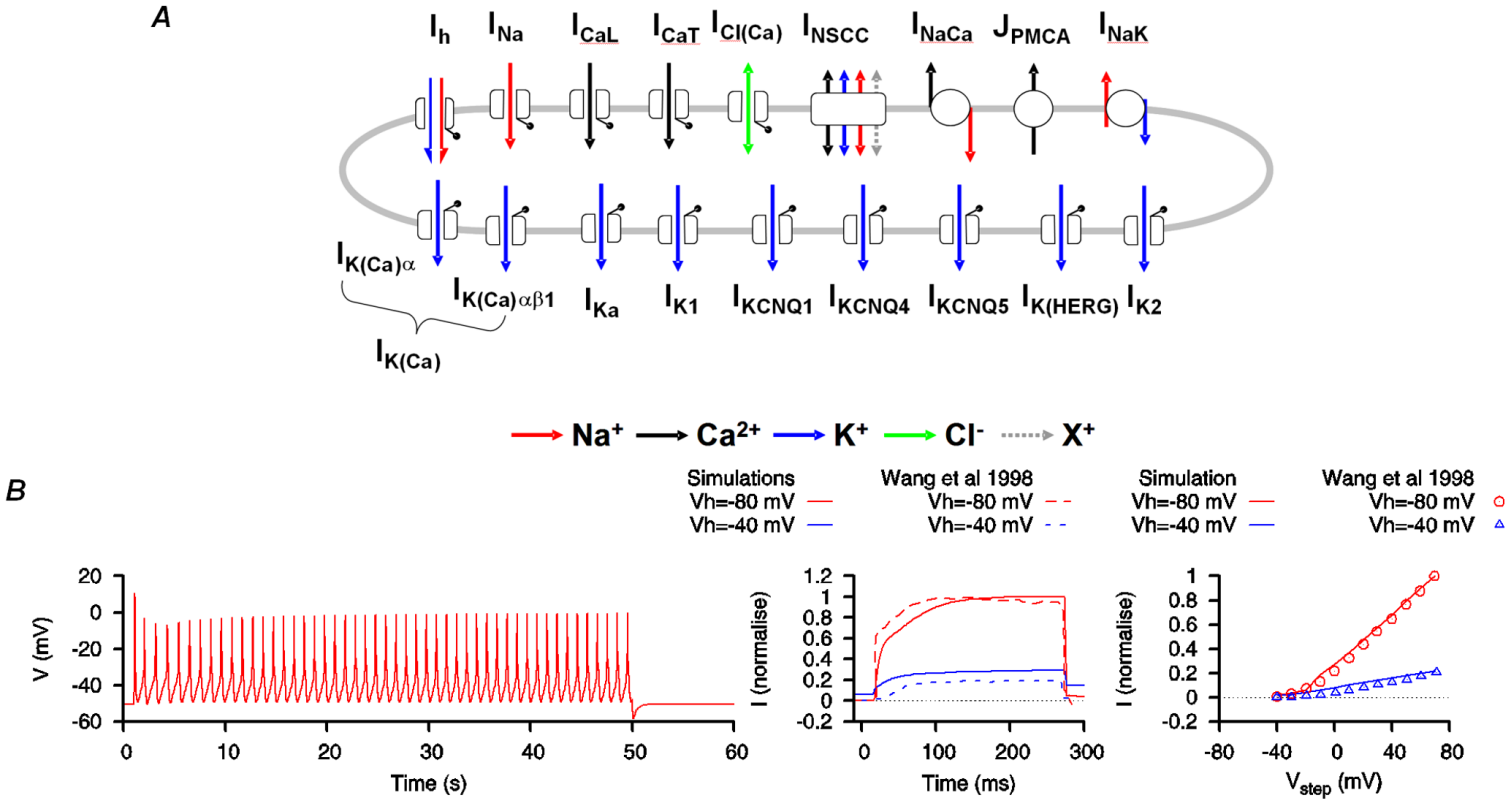
USMC model modification with incorporation of KCNQ1, KCNQ4, KCNQ5 and hERG currents. *A*, Effect of *I*_K1_ being partially replaced by a combination of *I*_KCNQ1_ (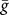
_KCNQ1_ =  0.0032 nS pF^−1^), *I*_KCNQ4_ (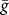
_KCNQ4_ =  0.024 nS pF^−1^) and *I*_hERG_ (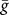
_hERG_ =  0.08 nS pF^−1^). Note that *I*_b_ has been replaced with *I*_KCNQ5_ (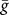
_KCNQ5_ =  0.016 nS pF^−1^). With this configuration, long-duration bursting APs were induced (left panel) while the whole cell K^+^ currents (middle panel) and the *I-V* relationships (right panel) matched the experimental data [44] (© Wang et al., 1998). *B*. Summary of the electrogenic components of the *modified USMC* model.

Given that uterine smooth muscle cells express components of KCNQ and hERG channels in a gestational dependent manner, we sought to test the proposition that the long duration bursting APs would be facilitated by the incorporation of a combination of *I*_KCNQ1_, *I*_KCNQ4_ and *I*_hERG_ in place of reduced *I*_K1_ (Figure 7A). Implementation of this scenario - with reduced *I*_K1_ (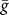
_K1_ =  0.24 nS pF^−1^), a combination of *I*_KCNQ1_ (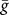
_KCNQ1_ =  0.0032 nS pF^−1^), *I*_KCNQ4_ (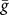
_KCNQ4_ =  0.024 nS pF^−1^) and *I*_hERG_ (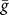
_hERG_ =  0.08 nS pF^−1^) and with *I*_b_ replaced by *I*_KCNQ5_ (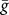
_KCNQ5_ =  0.016 nS pF^−1^) - accomplished our objective of reproducing the experimentally reported long-duration bursting AP forms [1]–[3]. Thus by incorporating biophysical details of *I*_KCNQ1_, *I*_KCNQ4_, *I*_KCNQ5_ and *I*_hERG_, these modifications corrected a main limitation of the original USMC model. It is likely that this was accomplished by strengthening the repolarization reserve [45] of the uterine cell thereby providing more ways to shape the USMC APs.

### Effects of changing hERG current conductance on USMC action potentials

**Figure 8:**
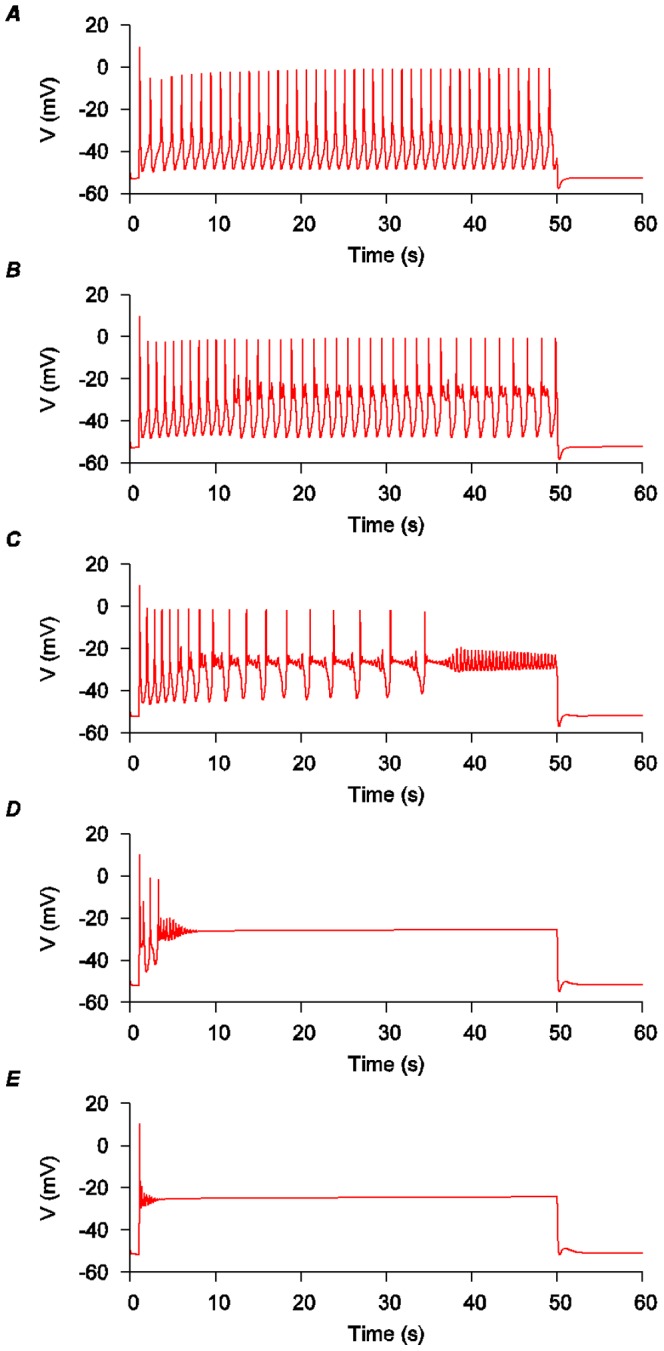
Effects of changing hERG current conductance on the *modified USMC* model AP form. *A*, The same conditions and AP form of the *modified USMC* model as depicted in Figure 7 wherein 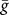
_hERG_ =  0.08 nS pF^−1^. *B-E*, the effects of progressively reducing *I*_hERG_ conductance. *B*, 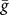
_hERG_ =  0.0464 nS pF^−1^; *C*, 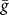
_hERG_ =  0.0224 nS pF^−1^; *D*, 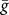
_hERG_ =  0.0064 nS pF^−1^; *E*, 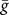
_hERG_ =  0.0008 nS pF^−1^.

Each of *I*_KCNQ1_, *I*_KCNQ4_ or *I*_hERG_ contributed to the ability of the *modified USMC* model to produce longer bursting APs. Of these, *I*_hERG_ appeared to exert the most potent influence in this regard (Figure S5). Indeed, in other electrically excitable cells, *I*_hERG_ contributes to AP spike frequency adaptation [46]. It is noteworthy that hERG-like currents have been reported to be decreased in murine and human myometrial tissues at late pregnancy [13], [14] . Our simulations also showed that progressive reductions in *I*_hERG_ conductance changed the simulated AP form from that of the long duration bursting AP (Figure 8A) to a more tonic plateau-type AP (Figure 8B-E). This result is similar to the effect of dofetilide on human myometrial APs [14] and provides a validation for the *modified USMC* model.

### Potential effects of changing both KCNQ and hERG current conductances on USMC action potentials

Both KCNQ and hERG currents are present in cardiac muscles and each contributes to stable and timely repolarization [45]. In uterine tissues, the regulations of KCNQ and hERG currents, and/or their molecular components, appear different from each other. Currents sensitive to hERG inhibitors have been reported in uterine tissues in early to mid gestation but these are decreased close to term [13], [14]. In contrast, almost all KCNQ alpha subunits expressions were suppressed during early to mid-gestation and increased at late term [8]. These data suggest KCNQ and hERG currents/components are gestationally regulated in a different manner and raises the question what may the effect be of changing the contributions of each of these particular K^+^ channel conductances? We utilized simulations of the *modified USMC* model to examine this.

**Figure 9:**
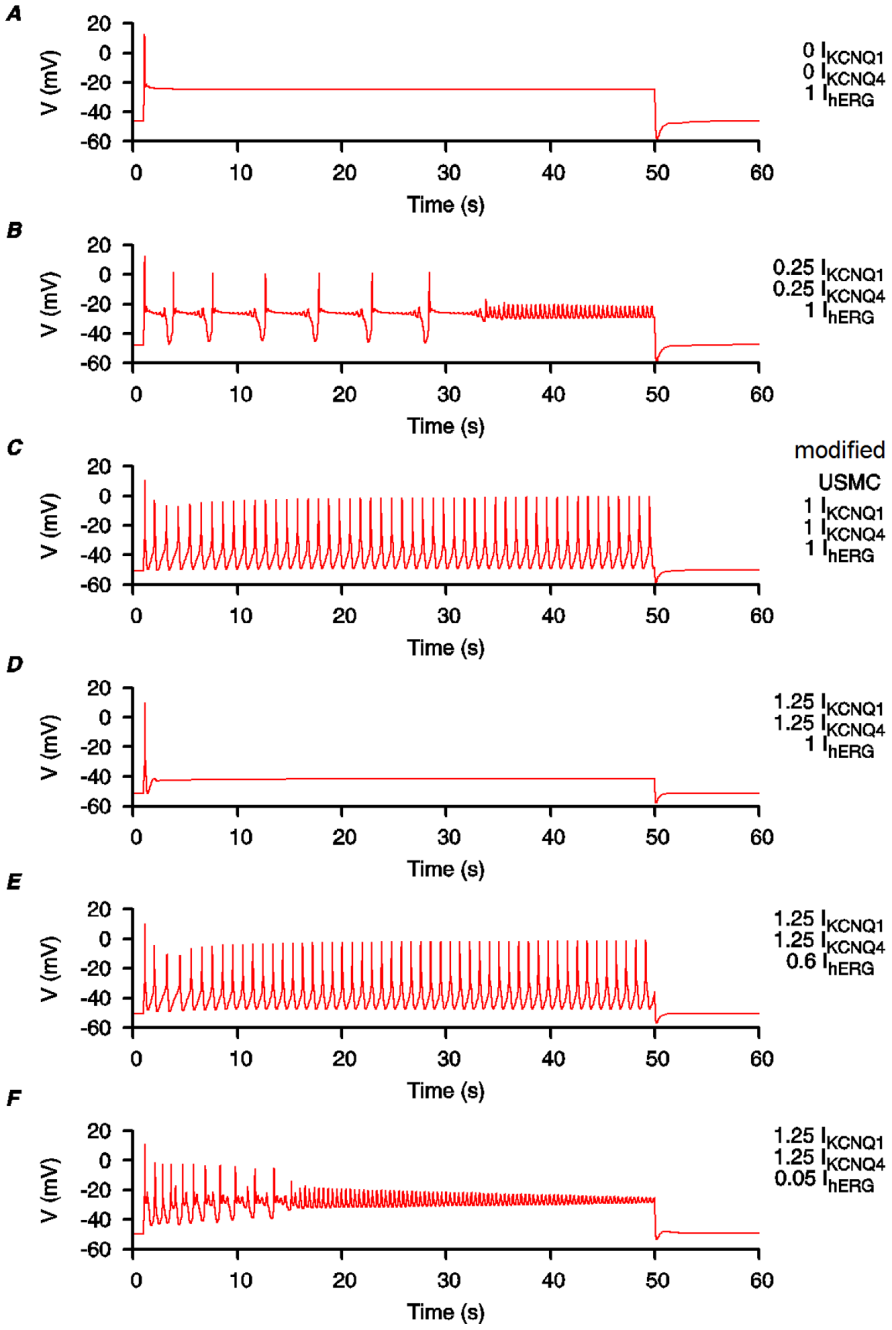
Effects of changing KCNQ and hERG current conductances on the *modified USMC* model AP form. Simulating the effects of increased expression of KCNQ subunits and reduction of HERG current in uterine smooth muscle cells during late term. The conductance values for *I*_KCNQ_ and *I*_hERG_ in the *modified USMC* model, as in Figure 7, are used as the reference values. Both *I*_KCNQ1_ and *I*_KCNQ4_ were changed by the same proportions. *A*, no *I*_KCNQ1_ or *I*_KCNQ4_; *I*_hERG_ at 1 fold. *B*, *I*_KCNQ1_ and *I*_KCNQ4_ at 0.25 fold; *I*_hERG_ at 1 fold. *C*, *I*_KCNQ1_, *I*_KCNQ4_ and *I*_hERG_ at 1 fold. *D*, *I*_KCNQ1_ and *I*_KCNQ4_ at 1.25 fold; *I*_hERG_ at 1 fold. *E*, *I*_KCNQ1_ and *I*_KCNQ4_ at 1.25 fold; *I*_hERG_ at 0.6 fold. *F*, *I*_KCNQ1_ and *I*_KCNQ4_ at 1.25 fold; *I*_hERG_ at 0.05 fold.

We began our simulations with no KCNQ currents but with full hERG current (Figure 9A). The result was a plateau-type AP with no initial bursting, qualitatively similar to the plateau-type APs recorded from uterine smooth muscle cells of mid-gestation pregnant rats [1]. We then examined the effects of either increasing *I*_KCNQ_ or reducing *I*_hERG_. With a little *I*_KCNQ_, but no change in *I*_hERG_, complex oscillations of membrane potentials began to develop around the plateau level (Figure 9B). With more *I*_KCNQ_, but still no change in *I*_hERG_, a bursting-type AP could be formed (Figure 9C), qualitatively similar to those bursting-type APs recorded from the uterine smooth muscle cells at end-of-term in rats [1]. Note that this is the configuration for our *modified USMC* model described in previous sections. Further increase of *I*_KCNQ_, and still no change in *I*_hERG_, suppressed the AP (Figure 9D). (As we do not know the maximum quantities of *I*_KCNQ_ in uterine smooth muscle cells, it is entirely possible for the *I*_KCNQ_ to exceed our reference (1.0) values). With *I*_KCNQ_ staying at the same level, but with reduced *I*_hERG_, the bursting-type AP can be formed again (Figure 9E). Further reduction of *I*_hERG_ suppressed the size of the bursting spikes and changed the AP into a complex form (Figure 9F). These simulations show that although *I*_KCNQ_ and *I*_hERG_ may be inversely regulated at late pregnancy, the alteration of conductances for each current potentially yields a fine tuning mechanism for uterine cells to develop and maintain long-duration bursting AP forms that are required for parturition.

### Conclusions and future considerations

We have identified *I*_KCNQ_ and *I*_hERG_ as important components for forming the characteristics long duration bursting-type APs of uterine smooth muscle cells. Also, the above computational approaches have enabled the establishment of a biophysically detailed mathematical model of uterine smooth muscle cell electrical excitability that reproduces the repertoire of experimentally-derived AP forms. In addition, this computational work alerts one to important biological features that would otherwise not be foreseen and can serve as an important tool for hypotheses generations and subsequent experimental testing. A key example is the model prediction that alterations of *I*_KCNQ_ and *I*_hERG_ have the potential to radically affect uterine AP forms and, as such, suggests that the physiological regulations of *I*_KCNQ_ and *I*_hERG_ may be crucial factors in determining labor onset and maintenance. Of note, there is increasing appreciation of the important roles that computational biology can make to improving our understanding of the complexities of uterine excitability and labor onset [47]–[48], to which the open-access *modified USMC* model herein looks to contribute by providing the research community with the opportunity to iteratively improve upon the biophysical detail of the components. This will include considerations of intracellular (e.g. metabolic-excitation-contraction coupling characteristics [49] and Ca^2+^ binding protein affinities) and intercellular (e.g. interstitial K^+^ accumulation [50]) mechanisms that may act on ionic currents to modulate AP form. To provide tissue-level models that encapsulate the whole process of excitation-contraction coupling one also has to consider spatio-temporal patterns of excitation [48]. Notwithstanding these longer-term research aspirations, our modified model of uterine cell excitability adds to our wider knowledge of the molecular and electrophysiological basis of cellular rhythms [51].

## Methods

The original USMC model from [4] describing 14 electrogenic membrane currents, intracellular calcium changes and associated force was used as the basis of this study. Detailed descriptions of the model equations and parameters values are given in [4].

This theoretical study was conducted in two parts. First, we performed an extensive one-at-a-time sensitivity analysis [52] on the USMC model kinetic properties to identify potential target parameters that can prolong or improve the initial bursting in an AP. Then, based on the results, we focused on including more kinetic details of the delay rectifying K^+^ currents. The USMC model is then modified by including four new voltage-gated K^+^ currents from the KCNQ and HERG families: *I*_KCNQ1_, *I*_KCNQ4_, *I*_KCNQ5_ and *I*_hERG_. The kinetics of these four K^+^ currents were described by Hodgkin-Huxley type formulations using published clonal data in various expression systems as there are no publish data from myometrial cells or tissues. The details of these currents are described in the Results and Discussion. The definitions of the symbols, the initial conditions and parameters values, the equations of the new potassium currents and the resulting *modified USMC* model are given in Table S1–3 and Appendix S1. The source code of the *modified USMC* model written in the C programming language is included in Appendix S2.

Unless otherwise stated, all simulated APs were evoked with a 50 s current clamp stimulus of −0.25 pA pF^−1^ at t = 1 s starting from the same initial conditions. For each maneuver of incorporating new currents into the cell model, the whole cell K^+^ currents and the corresponding *I-V* relationships were also constructed and validated against existing experimental data. Both the whole cell K^+^ currents and the *I-V* relationships were simulated by the same one-step voltage-clamp protocol: the membrane voltage was stepped up from a holding potential (*V*_h_) of either −40 mV or −80 mV to a step potential (*V*_step_) between -40 mV to 70 mV for 250 ms before stepping back to the *V*_h_. The model was started at the steady state conditions at *V*_h_ for each voltage-clamp step. For clarity, and to aid the readers comparison with the corresponding experimental time tracings from Wang et al. (1998) [44], only one simulated time tracing at *V*_step_ =  0 mV from each of the two *V*_h_ conditions were shown in our figures and Supplementary figures, and both the simulated and the extracted experimental time tracings were normalized to the maximal value from *V*_h_ =  −80 mV. The simulated *I-V* relationships were constructed from the maximum currents at each of the *V*_step_ normalized to the peak value at *V*_step_ =  70 mV from *V*_h_ =  −80 mV.

Simulations were computed with a fixed time step of 0.02 ms, using XPPAUT [53] with the fourth-order Runge-Kutta numerical integration method, in a IBM laptop PC with a Intel(R) Pentium(R) M 1.5 GHz single processor.

## Supporting Information

Figure S1
***I*** **_K1_ cannot be completely replaced by KCNQ1, KCNQ4, KCNQ5 or hERG current.** Effects of *I*_K1_ being completely replaced by either *I*_KCNQ1_, *I*_KCNQ4_, *I*_KCNQ5_ or *I*_hERG_. *A*, left hand panel: the standard configuration of the initial USMC model (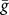
_K1_ =  0.52 nS pF^−1^), as depicted in Figure 1C, before *I*_K1_ replacement; middle panel: two simulated USMC whole cell K^+^ currents (*solid lines*) at a *V* step of 0 mV, one tracing was from a *V_h_* of -40 mV (*blue*) and another from a *V_h_* of -80 mV (*red*), and the corresponding experimental time tracings (*broken lines*) of Wang et al. (1998) [44] (© Wang et al., 1998); right hand panel: the simulated USMC K^+^ *I-V* relationships (*lines*) from both *V_h_* and the corresponding experimental data (*points*) of Wang et al., (1998) [44] (© Wang et al., 1998). B-E, the effects of *I*_K1_ being replaced by: *B*, *I*_KCNQ1_, *C*, *I*_KCNQ4_, *D*, *I*_KCNQ5_ and *E*, *I*_hERG_.(TIF)

Figure S2
**USMC model modification with reduced** ***I*** **_K1_ and added KCNQ1 current.** Effects of *I*_K1_ being partially replaced by *I*_KCNQ1_. *A*, the standard model configuration (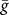
_K1_ =  0.52 nS pF^−1^) before *I*_K1_ replacement. Left hand panel: the standard AP simulation as depicted in Figure 1C; middle panel and right hand panel: the whole cell USMC *I-V* data. *B*-*E*, reduced *I*_K1_ (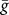
_K1_ =  0.24 nS pF^−1^) with different levels of KCNQ1 added: *B*, 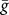
_KCNQ1_ =  0 nS pF^−1^; *C*, 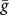
_KCNQ1_ =  0.028 nS pF^−1^; *D*, 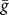
_KCNQ1_ =  0.032 nS pF^−1^; *E*, 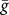
_KCNQ1_ =  0.036 nS pF^−1^.(TIF)

Figure S3
**USMC model modification with reduced** ***I*** **_K1_ and added KCNQ4 current.** Effects of *I*_K1_ being partially replaced by *I*_KCNQ4_. *A*, the standard model configuration (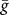
_K1_ =  0.52 nS pF^−1^) before *I*_K1_ replacement. Left hand panel: the standard AP simulation as depicted in Figure 1C; middle panel and right hand panel: the whole cell USMC *I-V* data. *B*-*E*, reduced *I*_K1_ (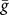
_K1_ =  0.24 nS pF^−1^) with different levels of KCNQ4 added: *B*, 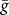
_KCNQ4_ =  0 nS pF^−1^; *C*, 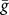
_KCNQ4_ =  0.03568 nS pF^−1^; *D*, 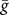
_KCNQ4_ =  0.0392 nS pF^−1^; *E*, 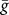
_KCNQ4_ =  0.04272 nS pF^−1^.(TIF)

Figure S4
**USMC model modification with reduced** ***I*** **_K1_ and added KCNQ5 current.** Effects of *I*_K1_ being partially replaced by *I*_KCNQ5_. *A*, the standard model configuration (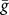
_K1_ =  0.52 nS pF^−1^) before *I*_K1_ replacement. Left hand panel: the standard AP simulation as depicted in Figure 1C; middle panel and right hand panel: the whole cell USMC *I-V* data. *B*-*E*, reduced *I*_K1_ (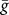
_K1_ =  0.24 nS pF^−1^) with different levels of KCNQ5 added: *B*, 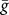
_KCNQ5_ =  0 nS pF^−1^; *C*, 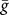
_KCNQ5_ =  0.0192 nS pF^−1^; *D*, 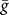
_KCNQ5_ =  0.02 nS pF^−1^; *E*, 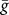
_KCNQ5_ =  0.0208 nS pF^−1^.(TIF)

Figure S5
**USMC model modification with reduced** ***I*** **_K1_ and added hERG current.** Effects of *I*_K1_ being partially replaced by *I*_hERG_. *A*, the standard model configuration (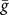
_K1_ =  0.52 nS pF^−1^) before *I*_K1_ replacement. Left hand panel: the standard AP simulation as depicted in Figure 1C; middle panel and right hand panel: the whole cell USMC *I-V* data. *B*-*E*, reduced *I*_K1_ (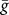
_K1_ =  0.24 nS pF^−1^) with different levels of hERG added: *B*, 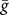
_hERG_ = 0 nS pF^−1^; *C*, 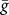
_hERG_ =  0.112 nS pF^−1^; *D*, 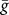
_hERG_ =  0.144 nS pF^−1^; *E*, 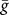
_hERG_ =  0.176 nS pF^−1^.(TIF)

Table S1 **Definitions of the equation symbols.**
(PDF)

Table S2 **Initial values of the dynamical variables used for the** ***modified USMC*** **model.**
(PDF)

Table S3 **Constant parameter values used for the** ***modified USMC*** **model.**
(PDF)

Appendix S1 **Equations used for the** ***modified USMC*** **model.**
(PDF)

Appendix S2 **Source codes in C for the** ***modified USMC*** **model.**
(BZ2)

## References

[1] Wilde DW, Marshall JM (1988) Effects of tetraethylammonium and 4-aminopyridine on the plateau potential of circular myometrium from the pregnant rat. Biol Reprod 38:836–845. 2456792 10.1095/biolreprod38.4.836

[2] Inoue Y, Okabe K, Soeda H (1999) Augmentation and suppression of action potentials by estradiol in the myometrium of pregnant rat. Can J Physiol Pharmacol 77:447–453. 10537231

[3] Khan RN, Smith SK, Morrison JJ, Ashford ML (1997) Ca^2+^ dependence and pharmacology of large-conductance K^+^ channels in nonlabor and labor human uterine myocytes. Am J Physiol 273:C1721–C1731. 9374660 10.1152/ajpcell.1997.273.5.C1721

[4] Tong WC, Choi CY, Kharche S, Holden AV, Zhang H, et al (2011) A computational model of the ionic currents, Ca^2+^ dynamics and action potentials underlying contraction of isolated uterine smooth muscle. PLoS ONE 6:e18685. 21559514 10.1371/journal.pone.0018685 PMC3084699

[5] Knock GA, Smirnov SV, Aaronson PI (1999) Voltage-gated K^+^ currents in freshly isolated myocytes of the pregnant human myometrium. J Physiol 518:769–781. 10420013 10.1111/j.1469-7793.1999.0769p.x PMC2269461

[6] Boyle MB, MacLusky NJ, Naftolin F, Kaczmarek LK (1987) Hormonal regulation of K^+^-channel messenger RNA in rat myometrium during oestrus cycle and in pregnancy. Nature 330:373–375. 2446134 10.1038/330373a0

[7] Boyle MB, Azhderian EM, MacLusky NJ, Naftolin F, Kaczmarek LK (1987) *Xenopus oocytes* injected with rat uterine RNA express very slowly activating potassium currents. Science 235:1221–1224. 2434999 10.1126/science.2434999

[8] McCallum LA, Pierce SL, England SK, Greenwood IA, Tribe RM (2011) The contribution of Kv7 channels to pregnant mouse and human myometrial contractility. J Cell Mol Med 15:577–586. 20132415 10.1111/j.1582-4934.2010.01021.x PMC3922379

[9] Bendahhou S, Marionneau C, Haurogne K, Larroque MM, Derand R, et al (2005) *In vitro* molecular interactions and distribution of KCNE family with KCNQ1 in the human heart. Cardiovasc Res 67:529–538. 16039274 10.1016/j.cardiores.2005.02.014

[10] Yeung SYM, Greenwood IA (2005) Electrophysiological and functional effects of the KCNQ channel blocker XE991 on murine portal vein smooth muscle cells. Br J Pharmacol 146:585–595. 16056238 10.1038/sj.bjp.0706342 PMC1751185

[11] Hadley JK, Noda M, Selyanko AA, Wood IC, Abogadie FC, et al (2000) Differential tetraethylammonium sensitivity of KCNQ1-4 potassium channels. Br J Pharmacol 129:413–415. 10711337 10.1038/sj.bjp.0703086 PMC1571869

[12] Robbins J (2001) KCNQ potassium channels: physiology, pathophysiology, and pharmacology. Pharmacol Ther 90:1–19. 11448722 10.1016/s0163-7258(01)00116-4

[13] Greenwood IA, Yeung SY, Tribe RM, Ohya S (2009) Loss of functional K^+^ channels encoded by ether-*à*-go-go-related genes in mouse myometrium prior to labour onset. J Physiol 587:2313–2326. 19332483 10.1113/jphysiol.2009.171272 PMC2697300

[14] Parkington HC, Stevenson J, Tonta MA, Paul J, Butler T, et al (2014) Diminished hERG K^+^ channel activity facilitates strong human labour contractions but is dysregulated in obese women. Nat Commun 17 5:4108. 10.1038/ncomms5108 24937480

[15] Sanguinetti MC, Jiang C, Curran ME, Keating MT (1995) A mechanistic link between an inherited and an acquired cardiac arrhythmia: HERG encodes the IKr potassium channel. Cell 81:299–307. 7736582 10.1016/0092-8674(95)90340-2

[16] Shoeb F, Malykhina AP, Akbarali HI (2003) Cloning and functional characterization of the smooth muscle ether-*à*-go-go-related gene K^+^ channel. potential role of a conserved amino acid substitution in the S4 region. J Biol Chem 278:2503–2514. 12427763 10.1074/jbc.M208525200

[17] Larsen AP, Olesen SP, Grunnet M, Jespersen T (2008) Characterization of hERG1a and hERG1b potassium channels-a possible role for hERG1b in the I(Kr) current. Pflugers Arch. 456(6):1137–48. 10.1007/s00424-008-0476-7 18504605

[18] Wang S, Morales MJ, Liu S, Strauss HC, Rasmusson RL (1996) Time, voltage and ionic concentration dependence of rectification of h-erg expressed in *Xenopus oocytes*. FEBS Lett. 389(2):167–73. 10.1016/0014-5793(96)00570-4 8766823

[19] Aydar E, Palmer C (2006) Expression and functional characterization of the human ether-*à*-go-go-related gene (HERG) K^+^ channel cardiac splice variant in *Xenopus laevis* oocytes. J Membr Biol 211:115–126. 17041783 10.1007/s00232-006-0010-9

[20] Kupershmidt S, Snyders DJ, Raes A, Roden DM (1998) A K^+^ channel splice variant common in human heart lacks a C-terminal domain required for expression of rapidly activating delayed rectifier current. J Biol Chem 273:27231–27235. 9765245 10.1074/jbc.273.42.27231

[21] Yeung SY, Greenwood IA (2007) Pharmacological and biophysical isolation of K^+^ currents encoded by ether-à-go-go-related genes in murine hepatic portal vein smooth muscle cells. Am J Physiol Cell Physiol. 292(1):C468–76. 10.1152/ajpcell.00142.2006 16870833

[22] London B, Trudeau MC, Newton KP, Beyer AK, Copeland NG, et al (1997) Two isoforms of the mouse ether-*à*-go-go-related gene coassemble to form channels with properties similar to the rapidly activating component of the cardiac delayed rectifier K^+^ current. Circ Res 81:870–878. 9351462 10.1161/01.res.81.5.870

[23] Tristani-Firouzi M, Sanguinetti MC (1998) Voltage-dependent inactivation of the human K^+^ channel KvLQT1 is eliminated by association with minimal K^+^ channel (minK) subunits. J Physiol 510(1):37–45. 9625865 10.1111/j.1469-7793.1998.037bz.x PMC2231024

[24] Franqueza L, Lin M, Shen J, Splawski I, Keating MT, et al (1999) Long QT syndrome-associated mutations in the S4-S5 linker of KvLQT1 potassium channels modify gating and interaction with minK subunits. J Biol Chem 274:21063–21070. 10409658 10.1074/jbc.274.30.21063

[25] Selyanko AA, Hadley JK, Wood IC, Abogadie FC, Jentsch TJ, et al (2000) Inhibition of KCNQ1-4 potassium channels expressed in mammalian cells via M1 muscarinic acetylcholine receptors. J Physiol 522(3):349–355. 10713961 10.1111/j.1469-7793.2000.t01-2-00349.x PMC2269765

[26] Pusch M, Ferrera L, Friedrich T (2001) Two open states and rate-limiting gating steps revealed by intracellular Na^+^ block of human KCNQ1 and KCNQ1/KCNE1 K^+^ channels. J Physiol 533:135–143. 11351022 10.1111/j.1469-7793.2001.0135b.x PMC2278592

[27] Seebohm G, Lerche C, Pusch M, Steinmeyer K, Brggemann A, et al (2001) A kinetic study on the stereospecific inhibition of KCNQ1 and I(Ks) by the chromanol 293B. Br J Pharmacol 134:1647–1654. 11739240 10.1038/sj.bjp.0704421 PMC1572901

[28] Peretz A, Schottelndreier H, Aharon-Shamgar LB, Attali B (2002) Modulation of homomeric and heteromeric KCNQ1 channels by external acidification. J Physiol 545:751–766. 12482884 10.1113/jphysiol.2002.028381 PMC2290713

[29] Gibor G, Yakubovich D, Peretz A, Attali B (2004) External barium affects the gating of KCNQ1 potassium channels and produces a pore block via two discrete sites. J Gen Physiol 124:83–102. 15226366 10.1085/jgp.200409068 PMC2229603

[30] Seebohm G, Strutz-Seebohm N, Baltaev R, Korniychuk G, Knirsch M, et al (2005) Regulation of KCNQ4 potassium channel prepulse dependence and current amplitude by SGK1 in *Xenopus* oocytes. Cell Physiol Biochem 16:255–262. 16301825 10.1159/000089851

[31] Bentzen BH, Schmitt N, Calloe K, Brown WD, Grunnet M, et al (2006) The acrylamide (S)-1 differentially affects Kv7 (KCNQ) potassium channels. Neuropharmacology 51:1068–1077. 16904708 10.1016/j.neuropharm.2006.07.001

[32] Jensen HS, Grunnet M, Olesen SP (2007) Inactivation as a new regulatory mechanism for neuronal Kv7 channels. Biophys J 92:2747–2756. 17237198 10.1529/biophysj.106.101287 PMC1831682

[33] Gao Z, Xiong Q, Sun H, Li M (2008) Desensitization of chemical activation by auxiliary subunits: convergence of molecular determinants critical for augmenting KCNQ1 potassium channels. J Biol Chem 283:22649–22658. 18490447 10.1074/jbc.M802426200 PMC2504881

[34] Blom SM, Schmitt N, Jensen HS (2009) The acrylamide (S)-2 as a positive and negative modulator of Kv7 channels expressed in *Xenopus laevis* oocytes. PLoS ONE 4:e8251. 20011514 10.1371/journal.pone.0008251 PMC2788219

[35] Larsen AP, Steffensen AB, Grunnet M, Olesen SP (2011) Extracellular potassium inhibits Kv7.1 potassium channels by stabilizing an inactivated state. Biophys J 101:818–827. 21843472 10.1016/j.bpj.2011.06.034 PMC3175082

[36] Yeung SYM, Lange W, Schwake M, Greenwood IA (2008a) Expression profile and characterisation of a truncated KCNQ5 splice variant. Biochem Biophys Res Commun 371:741–746. 18457656 10.1016/j.bbrc.2008.04.129

[37] Su TR, Chen CH, Huang SJ, Lee CY, Su MC, et al (2009) Functional study of the effect of phosphatase inhibitors on KCNQ4 channels expressed in *Xenopus* oocytes. Acta Pharmacol Sin 30:1220–1226. 19701239 10.1038/aps.2009.117 PMC4007189

[38] Soh H, Tzingounis AV (2010) The specific slow afterhyperpolarization inhibitor UCL2077 is a subtype-selective blocker of the epilepsy associated KCNQ channels. Mol Pharmacol 78:1088–1095. 20843955 10.1124/mol.110.066100 PMC2993466

[39] Brueggemann LI, Mackie AR, Martin JL, Cribbs LL, Byron KL (2011) Diclofenac distinguishes among homomeric and heteromeric potassium channels composed of KCNQ4 and KCNQ5 subunits. Mol Pharmacol 79:10–23. 20876743 10.1124/mol.110.067496 PMC3014280

[40] Lerche C, Scherer CR, Seebohm G, Derst C, Wei AD, et al (2000) Molecular cloning and functional expression of KCNQ5, a potassium channel subunit that may contribute to neuronal M-current diversity. J Biol Chem 275:22395–22400. 10787416 10.1074/jbc.M002378200

[41] Yeung S, Schwake M, Pucovsk V, Greenwood I (2008b) Bimodal effects of the Kv7 channel activator retigabine on vascular K+ currents. Br J Pharmacol 155:62–72. 18536747 10.1038/bjp.2008.231 PMC2527845

[42] Roura-Ferrer M, Etxebarria A, Sol L, Oliveras A, Comes N, et al (2009) Functional implications of KCNE subunit expression for the Kv7.5 (KCNQ5) channel. Cell Physiol Biochem 24:325–334. 19910673 10.1159/000257425

[43] Zhang S, Kehl SJ, Fedida D (2003) Modulation of human ether-*à*-go-go-related K^+^ (HERG) channel inactivation by Cs^+^ and K^+^ . J Physiol 548:691–702. 12626667 10.1113/jphysiol.2003.039198 PMC2342897

[44] Wang SY, Yoshino M, Sui JL, Wakui M, Kao PN, et al (1998) Potassium currents in freshly dissociated uterine myocytes from nonpregnant and late-pregnant rats. J Gen Physiol 112:737–756. 9834143 10.1085/jgp.112.6.737 PMC2229446

[45] Varró A, Baczkó I (2011) Cardiac ventricular repolarization reserve: a principle for understanding drug-related proarrhythmic risk. Br J Pharmacol. 164(1):14–36. 10.1111/j.1476-5381.2011.01367.x PMC3171857 21545574

[46] Chiesa N, Rosati B, Arcangeli A, Olivotto M, Wanke E (1997) A novel role for HERG K^+^ channels: spike-frequency adaptation. J Physiol 501:313–318. 9192303 10.1111/j.1469-7793.1997.313bn.x PMC1159479

[47] Sharp GC, Saunders PTK, Norman JE (2013) Computer models to study uterine activation at labour. Mol Hum Reprod 19:711–717. 23778245 10.1093/molehr/gat043

[48] Pervolaraki E, Holden AV (2013) Spatiotemporal patterning of uterine excitation patterns in human labour. Biosystems 112:63–72. 23499819 10.1016/j.biosystems.2013.03.012

[49] Taggart MJ, Wray S (1998) Hypoxia and smooth muscle function: key regulatory events during metabolic stress. J Physiol. 509:315–325. 10.1111/j.1469-7793.1998.315bn.x PMC2230985 9575282

[50] Young RC, Goloman G (2013) Phasic oscillations of extracellular potassium (K_o_) in pregnant rat myometrium. PLoS ONE. 8(5):e65110. 10.1371/journal.pone.0065110 PMC3665820 23724127

[51] Goldbeter A, Gérard C, Gonze D, Leloup JC, Dupont G (2012) Systems biology of cellular rhythms. FEBS Lett 586:2955–2965. 22841722 10.1016/j.febslet.2012.07.041

[52] Hamby DM (1994) A review of techniques for parameter sensitivity analysis of environmental models. Environmental Monitoring and Assessment 32:135–154. 24214086 10.1007/BF00547132

[53] Ermentrout GB (2002) Simulating, analysing, and animating dynamical systems: a guide to XPPAUT for researchers and students. Philadelphia, US: society for Industrial and Applied Mathematics.

