# Supplementary figures and images for: Computational Modeling Reveals Key Contributions of KCNQ and hERG Currents to the Malleability of Uterine Action Potentials Underpinning Labor
Source: PLoS One. 2014 Dec 4;9(12):e114034. doi: 10.1371/journal.pone.0114034 (PMC4256391; doi:10.1371/journal.pone.0114034)

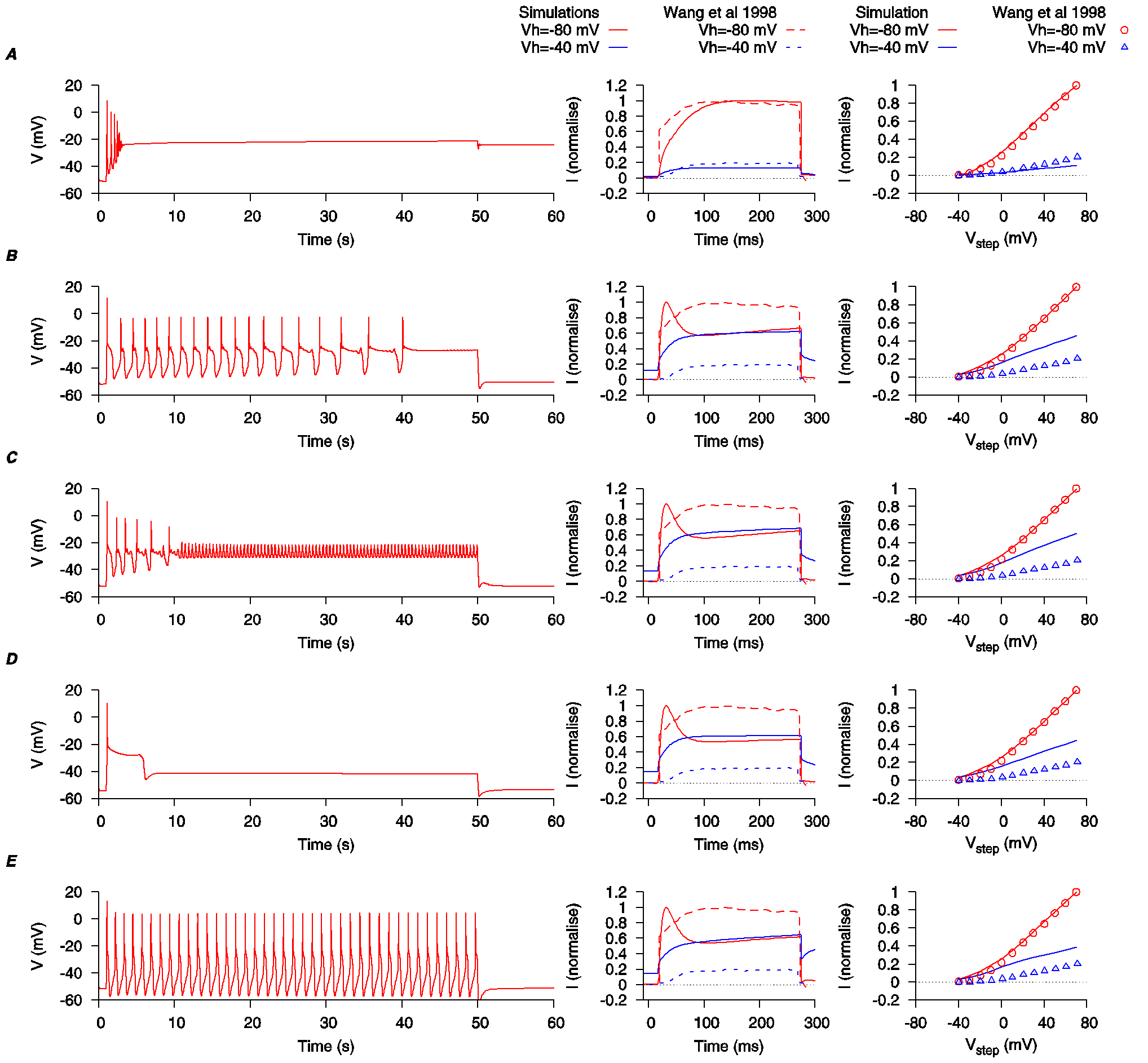

Supplement: Figure S1 — I K1 cannot be completely replaced by KCNQ1, KCNQ4, KCNQ5 or hERG current. Effects of I K1 being completely replaced by either I KCNQ1, I KCNQ4, I KCNQ5 or I hERG. A, left hand panel: the standard configuration of the initial USMC model ( K1 = 0.52 nS pF−1), as depicted in Figure 1C, before I K1 replacement; middle panel: two simulated USMC whole cell K+ currents (solid lines) at a V step of 0 mV, one tracing was from a Vh of -40 mV (blue) and another from a Vh of -80 mV (red), and the corresponding experimental time tracings (broken lines) of Wang et al. (1998) [44] (© Wang et al., 1998); right hand panel: the simulated USMC K+ I-V relationships (lines) from both Vh and the corresponding experimental data (points) of Wang et al., (1998) [44] (© Wang et al., 1998). B-E, the effects of I K1 being replaced by: B, I KCNQ1, C, I KCNQ4, D, I KCNQ5 and E, I hERG. (TIF) [file pone.0114034.s001.tif]

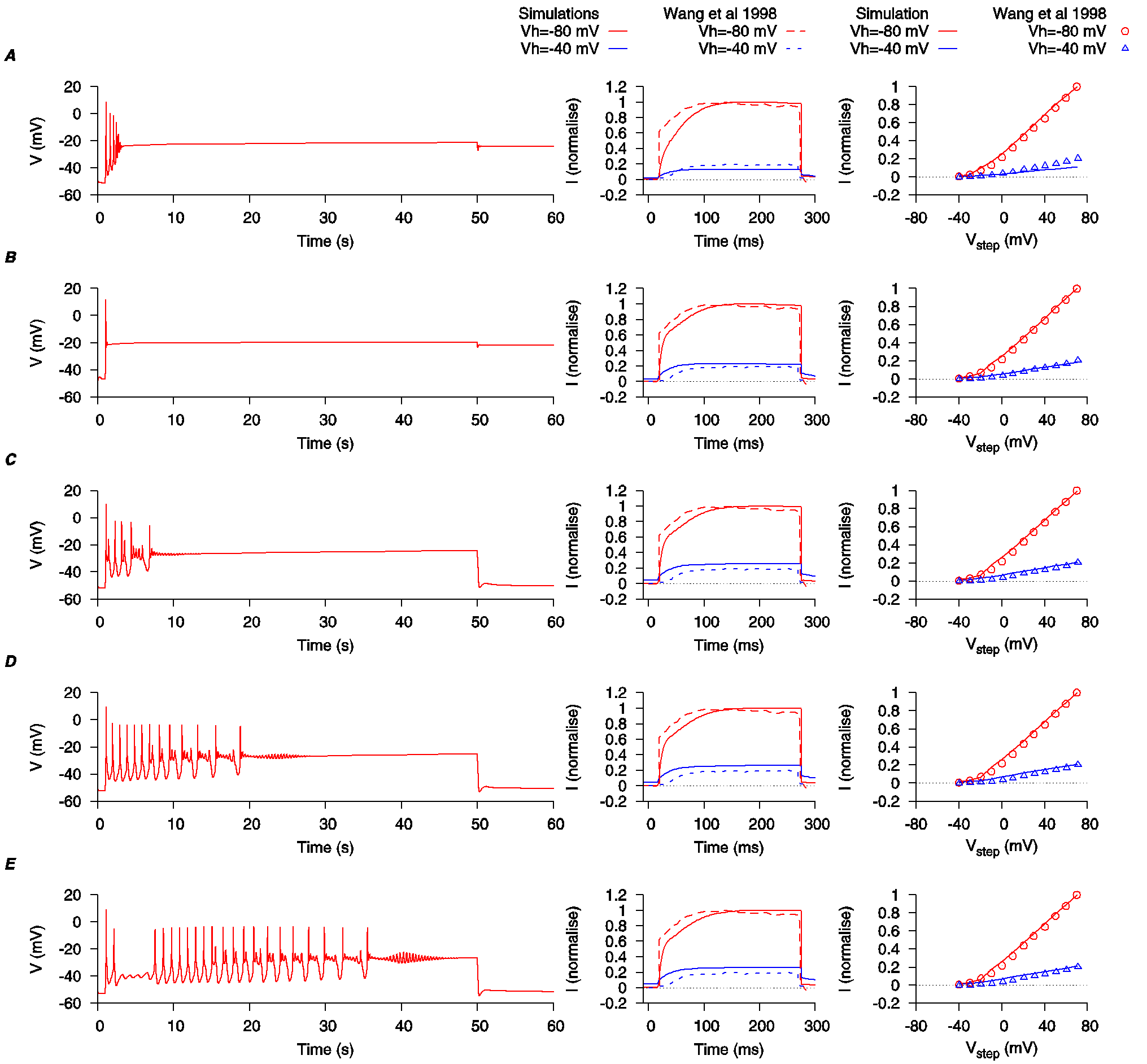

Supplement: Figure S2 — USMC model modification with reduced I K1 and added KCNQ1 current. Effects of I K1 being partially replaced by I KCNQ1. A, the standard model configuration ( K1 = 0.52 nS pF−1) before I K1 replacement. Left hand panel: the standard AP simulation as depicted in Figure 1C; middle panel and right hand panel: the whole cell USMC I-V data. B-E, reduced I K1 ( K1 = 0.24 nS pF−1) with different levels of KCNQ1 added: B, KCNQ1 = 0 nS pF−1; C, KCNQ1 = 0.028 nS pF−1; D, KCNQ1 = 0.032 nS pF−1; E, KCNQ1 = 0.036 nS pF−1. (TIF) [file pone.0114034.s002.tif]

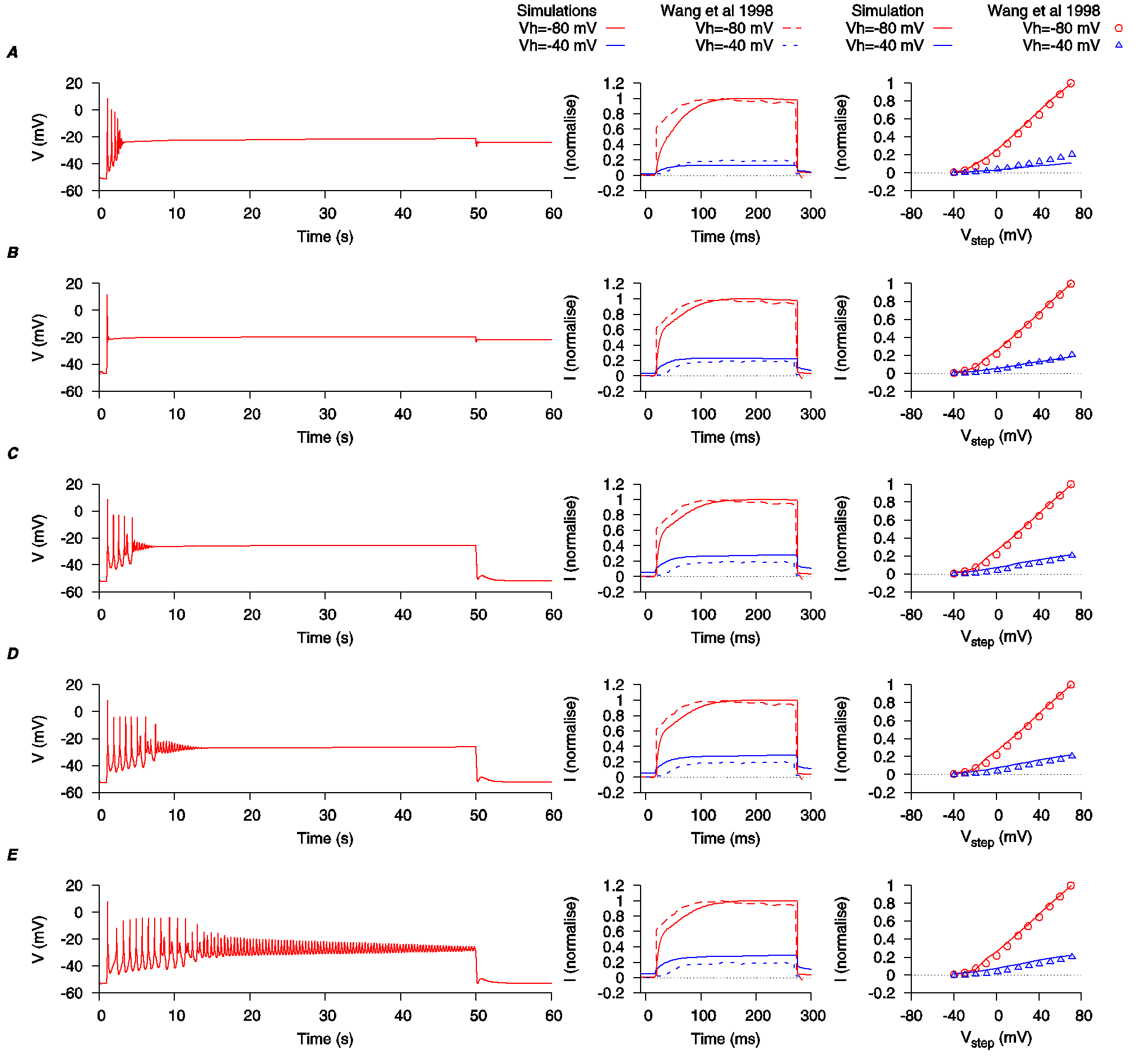

Supplement: Figure S3 — USMC model modification with reduced I K1 and added KCNQ4 current. Effects of I K1 being partially replaced by I KCNQ4. A, the standard model configuration ( K1 = 0.52 nS pF−1) before I K1 replacement. Left hand panel: the standard AP simulation as depicted in Figure 1C; middle panel and right hand panel: the whole cell USMC I-V data. B-E, reduced I K1 ( K1 = 0.24 nS pF−1) with different levels of KCNQ4 added: B, KCNQ4 = 0 nS pF−1; C, KCNQ4 = 0.03568 nS pF−1; D, KCNQ4 = 0.0392 nS pF−1; E, KCNQ4 = 0.04272 nS pF−1. (TIF) [file pone.0114034.s003.tif]

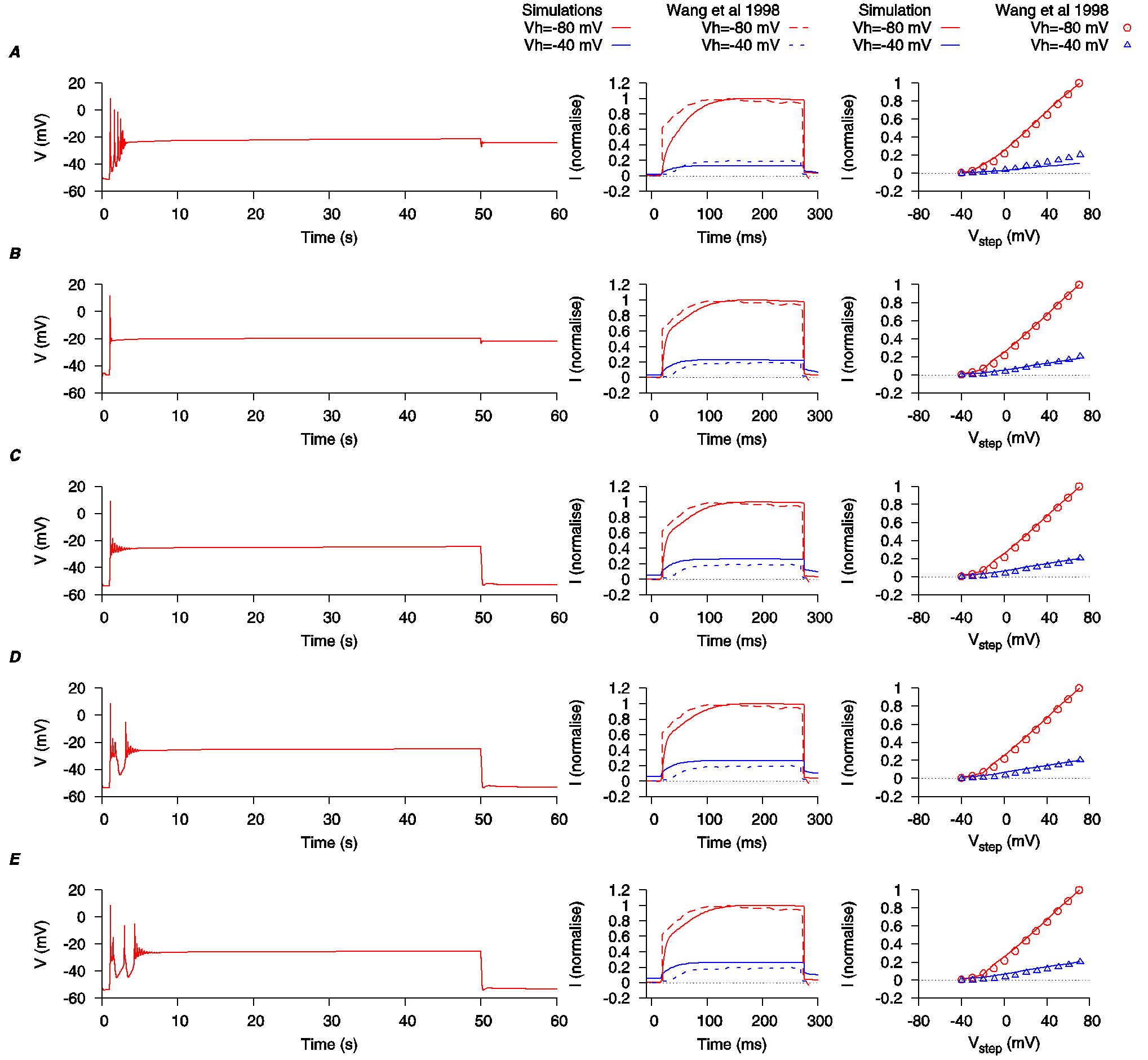

Supplement: Figure S4 — USMC model modification with reduced I K1 and added KCNQ5 current. Effects of I K1 being partially replaced by I KCNQ5. A, the standard model configuration ( K1 = 0.52 nS pF−1) before I K1 replacement. Left hand panel: the standard AP simulation as depicted in Figure 1C; middle panel and right hand panel: the whole cell USMC I-V data. B-E, reduced I K1 ( K1 = 0.24 nS pF−1) with different levels of KCNQ5 added: B, KCNQ5 = 0 nS pF−1; C, KCNQ5 = 0.0192 nS pF−1; D, KCNQ5 = 0.02 nS pF−1; E, KCNQ5 = 0.0208 nS pF−1. (TIF) [file pone.0114034.s004.tif]

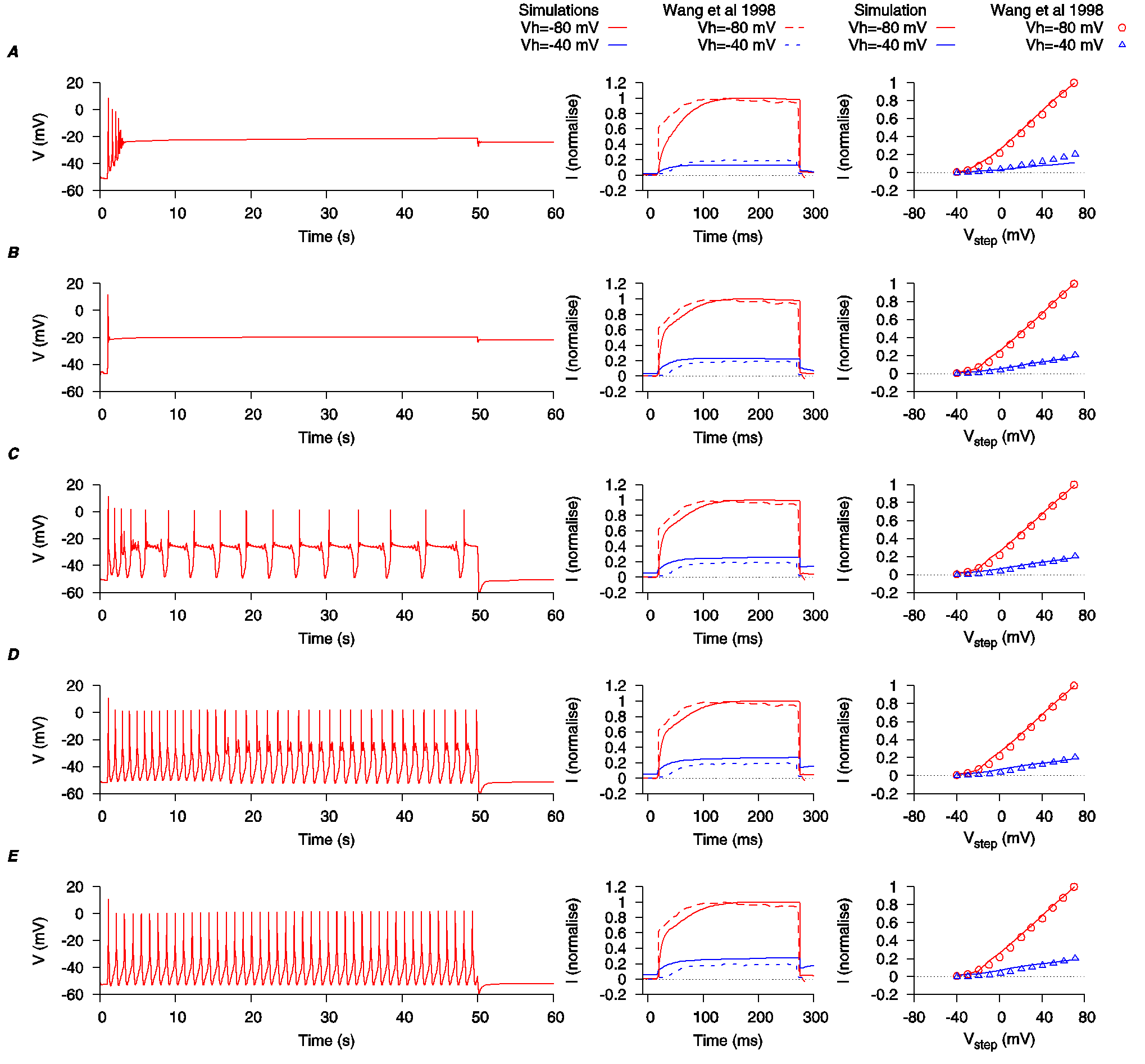

Supplement: Figure S5 — USMC model modification with reduced I K1 and added hERG current. Effects of I K1 being partially replaced by I hERG. A, the standard model configuration ( K1 = 0.52 nS pF−1) before I K1 replacement. Left hand panel: the standard AP simulation as depicted in Figure 1C; middle panel and right hand panel: the whole cell USMC I-V data. B-E, reduced I K1 ( K1 = 0.24 nS pF−1) with different levels of hERG added: B, hERG = 0 nS pF−1; C, hERG = 0.112 nS pF−1; D, hERG = 0.144 nS pF−1; E, hERG = 0.176 nS pF−1. (TIF) [file pone.0114034.s005.tif]
