# Supplementary material for: Computational Modeling Reveals Key Contributions of KCNQ and hERG Currents to the Malleability of Uterine Action Potentials Underpinning Labor
Source: PLoS One. 2014 Dec 4;9(12):e114034. doi: 10.1371/journal.pone.0114034 (PMC4256391; doi:10.1371/journal.pone.0114034)
Supplement: Table S1 — Definitions of the equation symbols. (PDF) [file pone.0114034.s006.pdf]

Table S1. Definitions of the equation symbols

Symbols	meaning
V	membrane potential
$V_{0.5}$	half (in)activation constant
V_h	holding potential
V_s	voltage steps
A_c	cell surface area
V_c	cell volume
C_{m}	specific membrane capacitance
R	the universal gas constant
F	the Faraday constant
T	absolute temperature
Ca^{2+}	calcium ion
Na^{+}	sodium ion
K^{+}	potassium ion
Cl^{-}	chloride ion
Mg^{2+}	magnesium ion
z_{Na}	Na^{+} valency
z_{Ca}	Ca^{2+} valency
z_{K}	K^{+} valency
β	Proportion of free Ca^{2+} ions
[X]i	intracellular concentration of ion X
[X]o	extracellular concentration of ion X
$[Ca^{2+}]_{\mathrm{i}}$	intracellular calcium
$[Ca^{2+}]_{\mathrm{o}}$	extracellular calcium
$[Na^{+}]_{\mathrm{i}}$	intracellular sodium
$[Na^{+}]_{\mathrm{o}}$	extracellular sodium
$[K^{+}]_{\mathrm{i}}$	intracellular potassium
$[K^{+}]_{\mathrm{o}}$	extracellular potassium
$[Cl^{-}]_{\mathrm{i}}$	intracellular chloride
$[Cl^{-}]_{\mathrm{o}}$	extracellular chloride
$[Mg^{2+}]_{\mathrm{o}}$	extracellular magnesium
E_{rev}	reversal potential
E_{CaL}	reversal potential of I_{CaL}
E_{CaT}	reversal potential of I_{CaT}
E_{Na}	reversal potential of I_{Na}
E_{h}	reversal potential of I_{h}
E_{K}	reversal potential of potassium currents
E_{Cl}	reversal potential of $I_{\mathrm{Cl(Ca)}}$
E_{NS}	reversal potential of I_{NSCC}
P_{Ca}	Permeability of calcium, ratio of flux concentration with respect to cesium
P'_{Ca}	Permeability of calcium, corrected with the effect of the electric field
P_{Na}	Permeability of sodium
P_{K}	Permeability of potassium
I	current
I_{ion}	total membrane ionic currents
I_{CaL}	L-type Ca^{2+} current

Continued on next page

Table S1 – continued from previous page

Symbols	meaning
I_{CaT}	T-type Ca^{2+} current
I_{Na}	fast inward Na^{+} current
I_{h}	hyperpolarization-activated current
I_{KCNQ1}	KCNQ1 K^{+} current
I_{KCNQ4}	KCNQ4 K^{+} current
I_{KCNQ5}	KCNQ5 K^{+} current
I_{hERG}	hERG K^{+} current
I_{K1}	voltage-gated K^{+} current, inactivate with $V_h \geq -40\,\mathrm{mV}$
I_{K2}	voltage-gated K^{+} current, inactivate with $V_h \geq 0\,\mathrm{mV}$
I_{Ka}	A-type transient K^{+} current
$I_{\mathrm{K(Ca)}}$	total Ca^{2+}-activated K^{+} current
I_{α}	$I_{\mathrm{K(Ca)}}$ subtype, consists of only α subunits
$I_{\alpha\beta 1}$	$I_{\mathrm{K(Ca)}}$ subtype, consists of α and $\beta 1$ subunits
$I_{\mathrm{Cl(Ca)}}$	Ca^{2+}-activated Cl^{-} currents
I_{NSCC}	non-specific cation current
$I_{\mathrm{NSCC,Ca}}$	calcium component of I_{NSCC}
I_{NaK}	Na^{+}-K^{+} pump current
I_{NaCa}	Na^{+}-Ca^{2+} exchanger current
J	Ca^{2+} flux
$J_{\mathrm{Ca,mem}}$	Ca^{2+} flux *via* membrane Ca^{2+} currents
J_{PMCA}	Ca^{2+} flux *via* plasma membrane Ca^{2+}-ATPase
J_{NaCa}	Ca^{2+} flux *via* Na^{+}-Ca^{2+} exchanger
$\bar{J}_{\mathrm{PMCA}}$	maximal J_{PMCA}
$\bar{g}$	maximum conductance
$\bar{g}_{\mathrm{CaL}}$	maximum conductance of I_{CaL}
$\bar{g}_{\mathrm{CaT}}$	maximum conductance of I_{CaT}
$\bar{g}_{\mathrm{Na}}$	maximum conductance of I_{Na}
$\bar{g}_{\mathrm{h}}$	maximum conductance of I_{h}
$\bar{g}_{\mathrm{KCNQ1}}$	maximum conductance of I_{KCNQ1}
$\bar{g}_{\mathrm{KCNQ4}}$	maximum conductance of I_{KCNQ4}
$\bar{g}_{\mathrm{KCNQ5}}$	maximum conductance of I_{KCNQ5}
$\bar{g}_{\mathrm{hERG}}$	maximum conductance of I_{hERG}
$\bar{g}_{\mathrm{K1}}$	maximum conductance of I_{K1}
$\bar{g}_{\mathrm{K2}}$	maximum conductance of I_{K2}
$\bar{g}_{\mathrm{Ka}}$	maximum conductance of I_{Ka}
$\bar{g}_{\mathrm{K(Ca)}}$	maximum conductance of $I_{\mathrm{K(Ca)}}$
$\bar{g}_{\mathrm{Cl(Ca)}}$	maximum conductance of $I_{\mathrm{Cl(Ca)}}$
$\bar{g}_{\mathrm{NS}}$	maximum conductance of I_{NSCC}
g_{NS}	conductance of I_{NSCC}
g_s	numerical constant, used for normalizing dependency of extracellular ions on the conductance of I_{NSCC}
$\bar{I}_{\mathrm{NaCa}}$	maximal I_{NaCa}
$\bar{I}_{\mathrm{NaK}}$	maximal I_{NaK}
$\bar{F}$	maximal force
n_{K}	Hill coefficient of $[K^{+}]_{o}$ dependency of I_{NaK}
n_{Na}	Hill coefficient of $[Na^{+}]_{i}$ dependency of I_{NaK}

Continued on next page

Table S1 – continued from previous page

Symbols	meaning
$\mathrm{n}_{\mathrm{PMCA}}$	Hill coefficient of plasma membrane Ca^{2+}-ATPase
$\mathrm{n}_{\mathrm{Allo}}$	Hill coefficient of allosteric regulation of Na^{+}-Ca^{2+} exchanger
n_{F}	Hill coefficient of the steady-state of calcium-dependent active force
f_{Ca}	Calcium inhibition of I_{CaL}
f_{Mg}	Magnesium inhibition of I_{NSCC}
f_{NaK}	Voltage-dependency of I_{NaK}
n_{NaK}	$[Na^{+}]_{\mathrm{i}}$ dependency of I_{NaK}
k_{NaK}	$[K^{+}]_{\mathrm{o}}$ dependency of I_{NaK}
k_{sat}	Saturation factor for Na^{+}-Ca^{2+} exchanger at very negative potential
$f_{1,NaCa}$	Potential dependency of I_{NaCa}
$f_{2,NaCa}$	Potential dependency of I_{NaCa}
f_{allo}	Allosteric regulation by $[Ca^{2+}]_{\mathrm{i}}$ of Na^{+}-Ca^{2+} exchanger
γ	Partition parameter of I_{NaCa}
p_a	proportion of I_{α} in $I_{\mathrm{K(Ca)}}$
p_b	proportion of $I_{\alpha\beta 1}$ in $I_{\mathrm{K(Ca)}}$
$K_{1,\mathrm{Cl}}$	Equilibrium constant for the calcium-binding property of c_{∞} of $I_{\mathrm{Cl(Ca)}}$
$K_{2,\mathrm{Cl}}$	Equilibrium constant for the voltage-dependency of c_{∞} of $I_{\mathrm{Cl(Ca)}}$
$K_{d,\mathrm{CaL}}$	Half-saturation concentration for $[Ca^{2+}]_{\mathrm{i}}$ inhibition of I_{CaL}
$K_{d,\mathrm{Mg}}$	Half-saturation concentration for $[Mg^{2+}]_{\mathrm{o}}$ inhibition of I_{NSCC}
$K_{m,\mathrm{K}}$	Half-saturation concentration for $[K^{+}]_{\mathrm{o}}$ dependency of I_{NaK}
$K_{m,\mathrm{Na}}$	Half-saturation concentration for $[Na^{+}]_{\mathrm{i}}$ dependency of I_{NaK}
$K_{m,\mathrm{PMCA}}$	Half-saturation concentration for $[Ca^{2+}]_{\mathrm{i}}$ association of plasma membrane Ca^{2+}-ATPase
$K_{m,\mathrm{Allo}}$	Half-saturation concentration for $[Ca^{2+}]_{\mathrm{i}}$ allosteric factor of Na^{+}-Ca^{2+} exchanger
$K_{m,\mathrm{Cai}}$	Dissociation constant for $[Ca^{2+}]_{\mathrm{i}}$ of Na^{+}-Ca^{2+} exchanger
$K_{m,\mathrm{Cao}}$	Dissociation constant for $[Ca^{2+}]_{\mathrm{o}}$ of Na^{+}-Ca^{2+} exchanger
$K_{m,\mathrm{Nai}}$	Dissociation constant for $[Na^{+}]_{\mathrm{i}}$ of Na^{+}-Ca^{2+} exchanger
$K_{m,\mathrm{Nao}}$	Dissociation constant for $[Na^{+}]_{\mathrm{o}}$ of Na^{+}-Ca^{2+} exchanger
$K_{m,\mathrm{F}}$	Apparent affinity constant for $[Ca^{2+}]_{\mathrm{i}}$ of contraction
b	Activation gate of I_{CaT}
c	Activation gate of $I_{\mathrm{Cl(Ca)}}$
d	Activation gate of I_{CaL}
f_1	Fast inactivation gate of I_{CaL}
f_2	Slow inactivation gate of I_{CaL}
g	Inactivation gate of I_{CaT}
h	Inactivation gate of I_{Na}
m	Activation gate of I_{Na}
p	Activation gate of I_{K2}
k_1	Fast inactivation gate of I_{K2}
k_2	Slow inactivation gate of I_{K2}
q	Activation gate of I_{K1}
r_1	Fast inactivation gate of I_{K1}
r_2	Slow inactivation gate of I_{K1}
$\mathrm{n}_{\mathrm{Q1f}}$	Fast activation gate of I_{KCNQ1}
$\mathrm{n}_{\mathrm{Q1s}}$	Slow activation gate of I_{KCNQ1}

Continued on next page

Table S1 – continued from previous page

Symbols	meaning
$\mathrm{w_{Q1}}$	Fast inactivation gate of I_{KCNQ1}
$\mathrm{s_{Q1}}$	Slow inactivation gate of I_{KCNQ1}
$\mathrm{n_{Q4}}$	Activation gate of I_{KCNQ4}
$\mathrm{s_{Q4}}$	Inactivation gate of I_{KCNQ4}
$\mathrm{n_{Q5f}}$	Fast activation gate of I_{KCNQ5}
$\mathrm{n_{Q5s}}$	Slow activation gate of I_{KCNQ5}
$\mathrm{w_{Q5}}$	Fast inactivation gate of I_{KCNQ5}
$\mathrm{s_{Q5}}$	Slow inactivation gate of I_{KCNQ5}
$\mathrm{h_{n1}}$	Fast activation gate of I_{hERG}
$\mathrm{h_{n2}}$	Slow activation gate of I_{hERG}
$\mathrm{h_s}$	Inactivation gate of I_{hERG}
s	Activation gate of I_{Ka}
x	Inactivation gate of I_{Ka}
$x_{\alpha\beta1}$	Activation gate of $I_{\alpha\beta1}$
x_{α}	Activation gate of I_{α}
y	Activation gate of I_{h}
ω	Activation gate of Force
b_∞	Steady-state of b
c_∞	Steady-state of c
d_∞	Steady-state of d
f_∞	Steady-state of f_1 and f_2
g_∞	Steady-state of g
h_∞	Steady-state of h
k_∞	Steady-state of k_1 and k_2
m_∞	Steady-state of m
p_∞	Steady-state of p
q_∞	Steady-state of q
r_∞	Steady-state of r_1 and r_2
$\mathrm{n_{Q1}}_\infty$	Steady-state of $\mathrm{n_{Q1f}}$ and $\mathrm{n_{Q1s}}$
$\mathrm{w_{Q1}}_\infty$	Steady-state of $\mathrm{w_{Q1}}$
$\mathrm{s_{Q1}}_\infty$	Steady-state of $\mathrm{s_{Q1}}$
$\mathrm{n_{Q4}}_\infty$	Steady-state of $\mathrm{n_{Q4}}$
$\mathrm{s_{Q4}}_\infty$	Steady-state of $\mathrm{s_{Q4}}$
$\mathrm{n_{Q5}}_\infty$	Steady-state of $\mathrm{n_{Q5f}}$ and $\mathrm{n_{Q5s}}$
$\mathrm{w_{Q5}}_\infty$	Steady-state of $\mathrm{w_{Q5}}$
$\mathrm{s_{Q5}}_\infty$	Steady-state of $\mathrm{s_{Q5}}$
$\mathrm{h_n}_\infty$	Steady-state of $\mathrm{h_{n1}}$ and $\mathrm{h_{n2}}$
$\mathrm{h_s}_\infty$	Steady-state of $\mathrm{h_s}$
s_∞	Steady-state of s
x_∞	Steady-state of x
y_∞	Steady-state of y
ω_∞	Steady-state of ω
$\alpha\beta1_\infty$	Steady-state of $x_{\alpha\beta1}$
α_∞	Steady-state of x_{α}
$V_{0.5,\alpha\beta1}$	Half activation of $x_{\alpha\beta1}$
$V_{0.5,\alpha}$	Half activation of x_{α}

Continued on next page

Table S1 – continued from previous page

Symbols	meaning
$z_{\alpha\beta 1}$	Gating charge of $x_{\alpha\beta 1}$
z_{α}	Gating charge of x_{α}
$\tau_{\alpha\beta 1}$	Time constant of $x_{\alpha\beta 1}$
τ_{α}	Time constant of x_{α}
τ_b	Time constant of b
τ_c	Time constant of c
τ_d	Time constant of d
τ_{f1}	Time constant of f_1
τ_{f2}	Time constant of f_2
τ_g	Time constant of g
τ_h	Time constant of h
τ_{k1}	Time constant of k_1
τ_{k2}	Time constant of k_2
τ_m	Time constant of m
τ_p	Time constant of p
τ_q	Time constant of q
τ_{r1}	Time constant of r_1
τ_{r2}	Time constant of r_2
τ_{nQ1f}	Time constant of $\mathrm{n_{Q1f}}$
τ_{nQ1s}	Time constant of $\mathrm{n_{Q1s}}$
τ_{wQ1}	Time constant of $\mathrm{w_{Q1}}$
τ_{sQ1}	Time constant of $\mathrm{s_{Q1}}$
τ_{nQ4}	Time constant of $\mathrm{n_{Q4}}$
τ_{sQ4}	Time constant of $\mathrm{s_{Q4}}$
τ_{nQ5f}	Time constant of $\mathrm{n_{Q5f}}$
τ_{nQ5s}	Time constant of $\mathrm{n_{Q5s}}$
τ_{wQ5}	Time constant of $\mathrm{w_{Q5f}}$
τ_{sQ5}	Time constant of $\mathrm{s_{Q5}}$
τ_{hn1}	Time constant of $\mathrm{h_{n1}}$
τ_{hn2}	Time constant of $\mathrm{h_{n2}}$
τ_{hs}	Time constant of $\mathrm{h_{s}}$
τ_s	Time constant of s
τ_x	Time constant of x
τ_y	Time constant of y
τ_{ω}	Time constant of ω
α_y	Rate constant of activation of y
β_y	Rate constant of deactivation of y
