# Supplementary material for: Computational Modeling Reveals Key Contributions of KCNQ and hERG Currents to the Malleability of Uterine Action Potentials Underpinning Labor
Source: PLoS One. 2014 Dec 4;9(12):e114034. doi: 10.1371/journal.pone.0114034 (PMC4256391; doi:10.1371/journal.pone.0114034)
Supplement: Table S2 — Initial values of the dynamical variables used for the modified USMC model. (PDF) [file pone.0114034.s007.pdf]

Table S2. Initial values of the dynamics variables used in model simulations.

Variables	Initial conditions
V	-50.81
$[Ca^{2+}]_i$	0.000124
m	0.166999
h	0.315608
b	0.585740
g	0.028175
d	0.016057
f_1	0.861723
f_2	0.861723
q	0.225602
r_1	0.126167
r_2	0.126167
n_{Q1f}	0.090437
n_{Q1s}	0.090437
w_{Q1}	0.923051
s_{Q1}	0.742674
n_{Q4}	0.108110
s_{Q4}	0.628062
n_{Q5f}	0.261889
n_{Q5s}	0.261889
w_{Q5}	0.923051
s_{Q5}	0.628062
h_{n1}	0.024990
h_{n2}	0.024990
h_s	0.529214
p	0.135875
k_1	0.994483
k_2	0.994483
x_α	0.000358
$x_{\alpha\beta 1}$	0.002674
s	0.045623
x	0.061628
y	0.001822
c	0.000670
ω	0.276830
