# Supplementary material for: Computational Modeling Reveals Key Contributions of KCNQ and hERG Currents to the Malleability of Uterine Action Potentials Underpinning Labor
Source: PLoS One. 2014 Dec 4;9(12):e114034. doi: 10.1371/journal.pone.0114034 (PMC4256391; doi:10.1371/journal.pone.0114034)
Supplement: Table S3 — Constant parameter values used for the modified USMC model. (PDF) [file pone.0114034.s008.pdf]

Table S3. Constant parameter values used in model simulations.

Parameters	values
T	308 K
F	96485 C mol^{-1}
R	8.314 J K^{-1} mol^{-1}
z_{Na}	1
z_K	1
z_{Ca}	2
$[Na^+]_i$	4 mM
$[Na^+]_o$	130 mM
$[K^+]_i$	140 mM
$[K^+]_o$	6 mM
$[Cl^-]_i$	46 mM
$[Cl^-]_o$	130 mM
$[Ca^{2+}]_o$	2.5 mM
$[Mg^{2+}]_o$	0.5 mM
C_m	1 μF cm^{-2}
A_c/V_c	4 cm^2 μl^{-1}
β	0.015
$\bar{J}_{\mathrm{PMCA}}$	3.5e^{-7} mM ms^{-1}
$K_{m,\mathrm{PMCA}}$	0.5 μM
n_{PMCA}	2
$\bar{I}_{\mathrm{NaCa}}$	5.63 pA pF^{-1}
$K_{m,\mathrm{Allo}}$	0.3 μM
n_{Allo}	4
k_{sat}	0.27
γ	0.35
$K_{m,\mathrm{Nai}}$	30.0 mM
$K_{m,\mathrm{Cai}}$	7 μM
$K_{m,\mathrm{Nao}}$	87.5 mM
$K_{m,\mathrm{Cao}}$	1.3 mM
$\bar{g}_{\mathrm{CaL}}$	0.6 nS pF^{-1}
E_{CaL}	45 mV
$K_{m,\mathrm{CaL}}$	1 μM
$\bar{g}_{\mathrm{CaT}}$	0.058 nS pF^{-1}
E_{CaT}	42 mV
$\bar{g}_{\mathrm{Na}}$	0 nS pF^{-1}
$\bar{g}_{\mathrm{h}}$	0.0542 nS pF^{-1}
$\bar{g}_{\mathrm{KCNQ1}}$	0.0032 nS pF^{-1}
$\bar{g}_{\mathrm{KCNQ4}}$	0.024 nS pF^{-1}
$\bar{g}_{\mathrm{KCNQ5}}$	0.016 nS pF^{-1}
$\bar{g}_{\mathrm{hERG}}$	0.08 nS pF^{-1}
$\bar{g}_{\mathrm{K1}}$	0.24 nS pF^{-1}
$\bar{g}_{\mathrm{K2}}$	0.032 nS pF^{-1}
$\bar{g}_{\mathrm{Ka}}$	0.16 nS pF^{-1}
$\bar{g}_{\mathrm{BK}}$	0.8 nS pF^{-1}
p_a	0.2

Continued on next page

Table S3 – continued from previous page

**Parameters**	**values**
p_b	0.1
$\bar{g}_{\mathrm{NS}}$	$0.0123\,\mathrm{nS\,pF^{-1}}$
$P_{\mathrm{Na}}/P_{\mathrm{K}}$	0.35
$P_{\mathrm{Ca}} : P_{\mathrm{Cs}}$	0.89
$P_{\mathrm{Na}} : P_{\mathrm{Cs}}$	0.9
$P_{\mathrm{K}} : P_{\mathrm{Cs}}$	1.3
$K_{d,\mathrm{Mg}}$	$0.28\,\mathrm{mM}$
$\bar{g}_{\mathrm{Cl}}$	$0.1875\,\mathrm{nS\,pF^{-1}}$
$\bar{I}_{\mathrm{NaK}}$	$1.7\,\mathrm{pA\,pF^{-1}}$
$K_{d,\mathrm{K}}$	$2.0\,\mathrm{mM}$
$\mathrm{n_K}$	1.5
$K_{m,\mathrm{Na}}$	$22.0\,\mathrm{mM}$
$\mathrm{n_{Na}}$	2
