# Supplementary material for: Computational Modeling Reveals Key Contributions of KCNQ and hERG Currents to the Malleability of Uterine Action Potentials Underpinning Labor
Source: PLoS One. 2014 Dec 4;9(12):e114034. doi: 10.1371/journal.pone.0114034 (PMC4256391; doi:10.1371/journal.pone.0114034)
Supplement: Appendix S1 — Equations used for the modified USMC model. (PDF) [file pone.0114034.s009.pdf]

Appendix S1. Equations used for the modified USMC model.

Equations for KCNQ1 current – I_{KCNQ1}

$$I_{\mathrm{KCNQ1}} = \bar{g}_{\mathrm{KCNQ1}}\ (0.3\ \mathrm{n_{Q1f}} + 0.7\ \mathrm{n_{Q1s}})\ \mathrm{w_{Q1}}\ \mathrm{s_{Q1}}\ (V - E_{\mathrm{K}}) \quad (1)$$

$$E_{\mathrm{K}} = \frac{RT}{F}\ln\left(\frac{[\mathrm{K}^+]_{\mathrm{o}}}{[\mathrm{K}^+]_{\mathrm{i}}}\right) \quad (2)$$

$$\mathrm{n_{Q1}}_{\infty} = \frac{1}{1+\exp\left(\frac{-(V+22.0)}{12.48}\right)} \quad (3)$$

$$\mathrm{w_{Q1}}_{\infty} = 0.49 + \frac{0.51}{1+\exp\left(\frac{V+1.084}{28.78}\right)} \quad (4)$$

$$\mathrm{s_{Q1}}_{\infty} = 0.34 + \frac{0.66}{1+\exp\left(\frac{V+45.3}{12.3}\right)} \quad (5)$$

$$\tau_{\mathrm{nQ1f}} = \frac{395.3}{1+\left(\frac{V+38.1}{33.59}\right)^2} \quad (6)$$

$$\tau_{\mathrm{nQ1s}} = 5503 - \frac{5345.4}{1+10^{(-0.02827(-23.9-V))}} - \frac{4590.6}{1+10^{(-0.0357(V+14.15))}} \quad (7)$$

$$\tau_{\mathrm{wQ1}} = 5.44 + \frac{29.2}{1+\left(\frac{V+48.09}{48.83}\right)^2} \quad (8)$$

$$\tau_{\mathrm{sQ1}} = 50000\ \mathrm{ms} \quad (9)$$

$$\frac{d\mathrm{n_{Q1f}}}{dt} = \frac{\mathrm{n_{Q1}}_{\infty} - \mathrm{n_{Q1f}}}{\tau_{\mathrm{nQ1f}}} \quad (10)$$

$$\frac{d\mathrm{n_{Q1s}}}{dt} = \frac{\mathrm{n_{Q1}}_{\infty} - \mathrm{n_{Q1s}}}{\tau_{\mathrm{nQ1s}}} \quad (11)$$

$$\frac{d\mathrm{w_{Q1}}}{dt} = \frac{\mathrm{w_{Q1}}_{\infty} - \mathrm{w_{Q1}}}{\tau_{\mathrm{wQ1}}} \quad (12)$$

$$\frac{d\mathrm{s_{Q1}}}{dt} = \frac{\mathrm{s_{Q1}}_{\infty} - \mathrm{s_{Q1}}}{\tau_{\mathrm{sQ1}}} \quad (13)$$

Equations for KCNQ4 current – I_{KCNQ4}

$$I_{\text{KCNQ4}} = \bar{g}_{\text{KCNQ4}}\ \text{n}_{\text{Q4}}\ \text{s}_{\text{Q4}}\ (V - E_{\text{K}}) \tag{14}$$

$$\text{n}_{\text{Q4}_\infty} = \frac{1}{1 + \exp\left(\dfrac{-(V + 15.04)}{16.95}\right)} \tag{15}$$

$$\text{s}_{\text{Q4}_\infty} = \frac{0.41}{1 + \exp\left(\dfrac{V + 86.84}{15.05}\right)} + \frac{0.59}{1 + \exp\left(\dfrac{V - 70.13}{13.37}\right)} \tag{16}$$

$$\tau_{\text{nQ4}} = 10 + \frac{895.9}{1 + \exp\left(\dfrac{-18.01 - V}{31.04}\right)} \tag{17}$$

$$\tau_{\text{sQ4}} = 1077 + \frac{185845}{1 + \left(\dfrac{V - 39.44}{7.34}\right)^2} \tag{18}$$

$$\frac{d\text{n}_{\text{Q4}}}{dt} = \frac{\text{n}_{\text{Q4}_\infty} - \text{n}_{\text{Q4}}}{\tau_{\text{nQ4}}} \tag{19}$$

$$\frac{d\text{s}_{\text{Q4}}}{dt} = \frac{\text{s}_{\text{Q4}_\infty} - \text{s}_{\text{Q4}}}{\tau_{\text{sQ4}}} \tag{20}$$

Equations for KCNQ5 current – I_{KCNQ5}

$$I_{\mathrm{KCNQ5}} = \bar{g}_{\mathrm{KCNQ5}}\ (0.2\ \mathrm{n}_{\mathrm{Q5f}} + 0.8\ \mathrm{n}_{\mathrm{Q5s}})\ \mathrm{w}_{\mathrm{Q5}}\ \mathrm{s}_{\mathrm{Q5}}\ (V - E_{\mathrm{K}}) \tag{21}$$

$$\mathrm{n}_{\mathrm{Q5}_\infty} = \frac{1}{1 + \exp\left(\dfrac{-(V + 36.55)}{13.76}\right)} \tag{22}$$

$$\mathrm{w}_{\mathrm{Q5}_\infty} = \mathrm{w}_{\mathrm{Q1}_\infty} \tag{23}$$

$$\mathrm{s}_{\mathrm{Q5}_\infty} = \mathrm{s}_{\mathrm{Q4}_\infty} \tag{24}$$

$$\tau_{\mathrm{nQ5f}} = 37.51 + \frac{539}{1 + \left(\dfrac{V + 40.24}{17.72}\right)^2} \tag{25}$$

$$\tau_{\mathrm{nQ5s}} = 1000\ \mathrm{ms} \tag{26}$$

$$\tau_{\mathrm{wQ5}} = \tau_{\mathrm{wQ1}} \tag{27}$$

$$\tau_{\mathrm{sQ5}} = \tau_{\mathrm{sQ4}} \tag{28}$$

$$\frac{d\mathrm{n}_{\mathrm{Q5f}}}{dt} = \frac{\mathrm{n}_{\mathrm{Q5}_\infty} - \mathrm{n}_{\mathrm{Q5f}}}{\tau_{\mathrm{nQ5f}}} \tag{29}$$

$$\frac{d\mathrm{n}_{\mathrm{Q5s}}}{dt} = \frac{\mathrm{n}_{\mathrm{Q5}_\infty} - \mathrm{n}_{\mathrm{Q5s}}}{\tau_{\mathrm{nQ5s}}} \tag{30}$$

$$\frac{d\mathrm{w}_{\mathrm{Q5}}}{dt} = \frac{\mathrm{w}_{\mathrm{Q5}_\infty} - \mathrm{w}_{\mathrm{Q5}}}{\tau_{\mathrm{wQ5}}} \tag{31}$$

$$\frac{d\mathrm{s}_{\mathrm{Q5}}}{dt} = \frac{\mathrm{s}_{\mathrm{Q5}_\infty} - \mathrm{s}_{\mathrm{Q5}}}{\tau_{\mathrm{sQ5}}} \tag{32}$$

Equations for hERG current – I_{hHERG}

$$I_{\mathrm{hERG}} = \bar{g}_{\mathrm{hERG}}(0.8\ \mathrm{h_{n1}} + 0.2\ \mathrm{h_{n2}})\ \mathrm{h_s}\ (V - E_{\mathrm{K}}) \quad (33)$$

$$\mathrm{h_{n\infty}} = \frac{1}{1+\exp\left(\dfrac{-(V+16)}{9.5}\right)} \quad (34)$$

$$\mathrm{h_{s\infty}} = \frac{1}{1+\exp\left(\dfrac{V+48}{24}\right)} \quad (35)$$

$$\tau_{\mathrm{hn1}} = 446.09 + \frac{1685.76}{\left(1+\exp\left(\dfrac{-(V+40.84)}{13.78}\right)\right)\left(1+\exp\left(\dfrac{V+20.63}{15.11}\right)\right)} \quad (36)$$

$$\tau_{\mathrm{hn2}} = 475.66 + \frac{16321.6}{\left(1+\exp\left(\dfrac{-(V+41.83)}{6.96}\right)\right)\left(1+\exp\left(\dfrac{V+23.24}{21.29}\right)\right)} \quad (37)$$

$$\tau_{\mathrm{hs}} = \frac{19.78}{1+\left(\dfrac{V+20.71}{44.28}\right)^2} - 0.378 \quad (38)$$

$$\frac{d\mathrm{h_{n1}}}{dt} = \frac{\mathrm{h_{n\infty}} - \mathrm{h_{n1}}}{\tau_{\mathrm{hn1}}} \quad (39)$$

$$\frac{d\mathrm{h_{n2}}}{dt} = \frac{\mathrm{h_{n\infty}} - \mathrm{h_{n2}}}{\tau_{\mathrm{hn2}}} \quad (40)$$

$$\frac{d\mathrm{h_s}}{dt} = \frac{\mathrm{h_{s\infty}} - \mathrm{h_s}}{\tau_{\mathrm{hs}}} \quad (41)$$

L-type Calcium current – I_{CaL}

$$I_{\mathrm{CaL}} = \bar{g}_{\mathrm{CaL}} d^2 f_{Ca} (0.8 f_1 + 0.2 f_2)(V - E_{\mathrm{CaL}}) \tag{42}$$

$$f_{Ca} = \frac{1}{1 + \left(\frac{[\mathrm{Ca}^{2+}]_{\mathrm{i}}}{K_{d,\mathrm{CaL}}} \right)^4} \tag{43}$$

$$d_\infty = \frac{1}{1 + \exp\left(\frac{-(V+22)}{7} \right)} \tag{44}$$

$$f_\infty = \frac{1}{1 + \exp\left(\frac{V+38}{7} \right)} \tag{45}$$

$$\tau_d = 2.29 + \frac{5.7}{1 + \left(\frac{V+29.97}{9} \right)^2} \tag{46}$$

$$\tau_{f1} = 12\mathrm{ms} \tag{47}$$

$$\tau_{f2} = 90.97 \left(1 - \frac{1}{\left(1 + \exp\left(\frac{V+13.96}{45.38} \right) \right) \left(1 + \exp\left(\frac{-(V+9.5)}{3.39} \right) \right)} \right) \tag{48}$$

$$\frac{dd}{dt} = \frac{d_\infty - d}{\tau_d} \tag{49}$$

$$\frac{df_1}{dt} = \frac{f_\infty - f_1}{\tau_{f1}} \tag{50}$$

$$\frac{df_2}{dt} = \frac{f_\infty - f_2}{\tau_{f2}} \tag{51}$$

Sodium current $- I_{\mathrm{Na}}$

$$I_{\mathrm{Na}} = \bar{g}_{\mathrm{Na}} m^3 h (V - E_{\mathrm{Na}}) \tag{52}$$

$$E_{\mathrm{Na}} = \frac{RT}{F} \ln\left(\frac{[\mathrm{Na}^+]_{\mathrm{o}}}{[\mathrm{Na}^+]_{\mathrm{i}}}\right) \tag{53}$$

$$m_\infty = \frac{1}{1 + \exp\left(\frac{-(V + 35.96)}{9.24}\right)} \tag{54}$$

$$h_\infty = \frac{1}{1 + \exp\left(\frac{V + 57}{8}\right)} \tag{55}$$

$$\tau_m = 0.25 + \frac{7}{1 + \exp\left(\frac{V + 38}{10}\right)} \tag{56}$$

$$\tau_h = 0.9 + \frac{1002.85}{1 + \left(\frac{V + 47.5}{1.5}\right)^2} \tag{57}$$

$$\frac{dm}{dt} = \frac{m_\infty - m}{\tau_m} \tag{58}$$

$$\frac{dh}{dt} = \frac{h_\infty - h}{\tau_h} \tag{59}$$

T-type Calcium current – I_{CaT}

$$I_{\mathrm{CaT}} = \bar{g}_{\mathrm{CaT}} b^2 g (V - E_{\mathrm{CaT}}) \tag{60}$$

$$b_\infty = \frac{1}{1+\exp\left(\dfrac{-(V+54.23)}{9.88}\right)} \tag{61}$$

$$g_\infty = 0.02 + \frac{0.98}{1+\exp\left(\dfrac{V+72.98}{4.64}\right)} \tag{62}$$

$$\tau_b = 0.45 + \frac{3.9}{1+\left(\dfrac{V+66}{26}\right)^2} \tag{63}$$

$$\tau_g = \left(150 - \frac{150}{\left(1+\exp\left(\dfrac{V-417.43}{203.18}\right)\right)\left(1+\exp\left(\dfrac{-(V+61.11)}{8.07}\right)\right)}\right) \tag{64}$$

$$\frac{db}{dt} = \frac{b_\infty - b}{\tau_b} \tag{65}$$

$$\frac{dg}{dt} = \frac{g_\infty - g}{\tau_g} \tag{66}$$

Hyperpolarisation-activated current $-\ I_{\rm h}$

$$I_{\rm h} = \bar{g}_{\rm h} y(V - E_{\rm h}) \tag{67}$$

$$E_{\rm h} = \frac{RT}{F}\ln\left(\frac{[{\rm K}^+]_{\rm o} + (P_{\rm Na}/P_{\rm K})[{\rm Na}^+]_{\rm o}}{[{\rm K}^+]_{\rm i} + (P_{\rm Na}/P_{\rm K})[{\rm Na}^+]_{\rm i}}\right) \tag{68}$$

$$y_\infty = \frac{1}{1 + \exp\left(\dfrac{V + 105.39}{8.66}\right)} \tag{69}$$

$$\tau_y = \frac{1}{3.5e^{-6}\exp\left(-0.0497V\right) + 0.04\exp\left(0.0521V\right)} \tag{70}$$

$$\frac{dy}{dt} = \frac{y_\infty - y}{\tau_y} \tag{71}$$

Voltage dependent potassium current $- I_{\mathrm{K1}}$

$$I_{\mathrm{K1}} = \bar{g}_{\mathrm{K1}} q^2 (0.38 r_1 + 0.62 r_2)(V - E_{\mathrm{K}}) \tag{72}$$

$$q_\infty = \frac{1}{1 + \exp\left(\dfrac{-(V + 18.67)}{26.66}\right)} \tag{73}$$

$$r_\infty = \frac{1}{1 + \exp\left(\dfrac{V + 63}{6.3}\right)} \tag{74}$$

$$\tau_q = \frac{500}{1 + \left(\dfrac{V + 60.71}{15.79}\right)^2} \tag{75}$$

$$\tau_{r1} = \frac{5e^4}{1 + \left(\dfrac{V + 62.71}{35.86}\right)^2} \tag{76}$$

$$\tau_{r2} = 3e^4 + \frac{2.2e^5}{1 + \exp\left(\dfrac{V + 22}{4}\right)} \tag{77}$$

$$\frac{dq}{dt} = \frac{q_\infty - q}{\tau_q} \tag{78}$$

$$\frac{dr_1}{dt} = \frac{r_\infty - r_1}{\tau_{r1}} \tag{79}$$

$$\frac{dr_2}{dt} = \frac{r_\infty - r_2}{\tau_{r2}} \tag{80}$$

Voltage dependent potassium current $-\ I_{\mathrm{K2}}$

$$I_{\mathrm{K2}} = \bar{g}_{\mathrm{K2}} p^2 (0.75 k_1 + 0.25 k_2)(V - E_{\mathrm{K}}) \tag{81}$$

$$p_\infty = \frac{1}{1 + \exp\left(\frac{-(V + 0.948)}{17.91}\right)} \tag{82}$$

$$k_\infty = \frac{1}{1 + \exp\left(\frac{(V + 21.2)}{5.7}\right)} \tag{83}$$

$$\tau_p = \frac{100}{1 + \left(\frac{V + 64.1}{28.67}\right)^2} \tag{84}$$

$$\tau_{k1} = 1e^6 \left(1 - \frac{1}{\left(1 + \exp\left(\frac{V - 315}{50}\right)\right)\left(1 + \exp\left(\frac{-(V + 74.9)}{8}\right)\right)}\right) \tag{85}$$

$$\tau_{k2} = 2.5e^6 \left(1 - \frac{1}{\left(1 + \exp\left(\frac{V - 132.87}{25.40}\right)\right)\left(1 + \exp\left(\frac{-(V + 24.92)}{2.68}\right)\right)}\right) \tag{86}$$

$$\frac{dp}{dt} = \frac{p_\infty - p}{\tau_p} \tag{87}$$

$$\frac{dk_1}{dt} = \frac{k_\infty - k_1}{\tau_{k1}} \tag{88}$$

$$\frac{dk_2}{dt} = \frac{k_\infty - k_2}{\tau_{k2}} \tag{89}$$

Transient potassium current – I_{Ka}

$$I_{\mathrm{Ka}} = \bar{g}_{\mathrm{Ka}} s x (V - E_{\mathrm{K}}) \tag{90}$$

$$s_\infty = \frac{1}{1 + \exp\left(\dfrac{-(V + 27.79)}{7.57}\right)} \tag{91}$$

$$x_\infty = 0.02 + \frac{0.98}{1 + \exp\left(\dfrac{V + 69.5}{6}\right)} \tag{92}$$

$$\tau_s = \frac{17}{1 + \left(\dfrac{V + 20.52}{35}\right)^2} \tag{93}$$

$$\tau_x = 7.5 + \frac{10}{1 + \left(\dfrac{V + 34.18}{120}\right)^2} \tag{94}$$

$$\frac{ds}{dt} = \frac{s_\infty - s}{\tau_s} \tag{95}$$

$$\frac{dx}{dt} = \frac{x_\infty - x}{\tau_x} \tag{96}$$

Calcium-activation potassium current $-$ $I_{\mathrm{K,Ca}}$

$$I_{\mathrm{K(Ca)}} = \bar{g}_{\mathrm{K(Ca)}}(p_a I_\alpha + p_b I_{\alpha\beta1}) \tag{97}$$

$$I_\alpha = x_\alpha(V - E_{\mathrm{K}}) \tag{98}$$

$$z_\alpha = \frac{8.38}{1+\left(\frac{1000[\mathrm{Ca}^{2+}]_{\mathrm{i}} + 1538.29}{739.06}\right)^2} - \frac{0.749}{1+\left(\frac{1000[\mathrm{Ca}^{2+}]_{\mathrm{i}} - 0.063}{0.162}\right)^2} \tag{99}$$

$$V_{0.5,\alpha} = \frac{5011.47}{1+\left(\frac{1000[\mathrm{Ca}^{2+}]_{\mathrm{i}} + 0.238}{0.000239}\right)^{0.423}} - 37.51 \tag{100}$$

$$x_{\alpha\infty} = \frac{1}{1+\exp\left(-\frac{z_\alpha F(V - V_{0.5,\alpha})}{RT}\right)} \tag{101}$$

$$\tau_\alpha = \frac{2.41}{1+\left(\frac{V - 158.78}{-52.15}\right)^2} \tag{102}$$

$$\frac{dx_\alpha}{dt} = \frac{x_{\alpha\infty} - x_\alpha}{\tau_\alpha} \tag{103}$$

$$I_{\alpha\beta1} = x_{\alpha\beta1}(V - E_{\mathrm{K}}) \tag{104}$$

$$z_{\alpha\beta1} = \frac{1.4}{1+\left(\frac{1000[\mathrm{Ca}^{2+}]_{\mathrm{i}} + 228.71}{684.95}\right)^2} - \frac{0.681}{1+\left(\frac{1000[\mathrm{Ca}^{2+}]_{\mathrm{i}} - 0.219}{0.428}\right)^2} \tag{105}$$

$$V_{0.5,\alpha\beta1} = \frac{8540.23}{1+\left(\frac{1000[\mathrm{Ca}^{2+}]_{\mathrm{i}} + 0.401}{0.00399}\right)^{0.668}} - 109.28 \tag{106}$$

$$x_{\alpha\beta1_\infty} = \frac{1}{1+\exp\left(-\frac{z_{\alpha\beta1} F(V - V_{0.5,\alpha\beta1})}{RT}\right)} \tag{107}$$

$$\tau_{\alpha\beta1} = \frac{13.8}{1+\left(\frac{V - 153.02}{66.5}\right)^2} \tag{108}$$

$$\frac{dx_{\alpha\beta1}}{dt} = \frac{x_{\alpha\beta1_\infty} - x_{\alpha\beta1}}{\tau_{\alpha\beta1}} \tag{109}$$

Calcium-activated chloride current – $I_{\mathrm{Cl(Ca)}}$

$$I_{\mathrm{Cl(Ca)}} = \bar{g}_{\mathrm{Cl}} c (V - E_{\mathrm{Cl}}) \tag{110}$$

$$E_{\mathrm{Cl}} = \frac{RT}{F} \ln \left(\frac{[\mathrm{Cl}^-]_{\mathrm{i}}}{[\mathrm{Cl}^-]_{\mathrm{o}}} \right) \tag{111}$$

$$K_{1,\mathrm{Cl}} = 0.0006 \exp \left(\frac{2.53FV}{RT} \right) \tag{112}$$

$$K_{2,\mathrm{Cl}} = 0.1 \exp \left(\frac{-5FV}{RT} \right) \tag{113}$$

$$c_{\infty} = \frac{1}{1 + K_{2,\mathrm{Cl}} \left(\left(\frac{K_{1,\mathrm{Cl}}}{[\mathrm{Ca}^{2+}]_{\mathrm{i}}} \right)^2 + \frac{K_{1,\mathrm{Cl}}}{[\mathrm{Ca}^{2+}]_{\mathrm{i}}} + 1 \right)} \tag{114}$$

$$\tau_c = \frac{210}{1 + \exp \left(\frac{V + 4.56}{11.62} \right)} + \frac{170}{1.0 + \exp \left(\frac{-(V + 25.5)}{11.62} \right)} - 160 \tag{115}$$

$$\frac{dc}{dt} = \frac{c_{\infty} - c}{\tau_c} \tag{116}$$

Non-selective cation current – I_{NSCC}

$$I_{\mathrm{NSCC}} = g_{\mathrm{NS}} f_{Mg} (V - E_{\mathrm{NS}}) \tag{117}$$

$$g_{\mathrm{NS}} = \bar{g}_{\mathrm{NS}} \left[0.5 g([\mathrm{Ca}^{2+}]_{\mathrm{o}}) + g([\mathrm{Na}^{+}]_{\mathrm{o}}) + 1.19 g([\mathrm{K}^{+}]_{\mathrm{o}})\right] + \bar{g}_{\mathrm{L}} \tag{118}$$

$$g([\mathrm{X}]_{\mathrm{o}}) = \frac{1}{g_s} \frac{0.03}{1 + \left(\dfrac{150}{[\mathrm{X}]_{\mathrm{o}} + 10^{-8}}\right)^2}, \quad g_s = \begin{cases} 0.000525 & \textbf{if ion is } \mathrm{Ca}^{2+} \\ 0.0123 & \textbf{otherwise} \end{cases} \tag{119}$$

$$f_{Mg} = 0.1 + \frac{0.9}{1 + \left(\dfrac{[\mathrm{Mg}^{2+}]_{\mathrm{o}}}{K_{d,\mathrm{Mg}}}\right)^{1.3}} \tag{120}$$

$$E_{\mathrm{NS}} = \frac{RT}{F} \ln \left(\frac{\dfrac{P_{\mathrm{Na}}}{P_{\mathrm{Cs}}}[\mathrm{Na}^{+}]_{\mathrm{o}} + \dfrac{P_{\mathrm{K}}}{P_{\mathrm{Cs}}}[\mathrm{K}^{+}]_{\mathrm{o}} + \dfrac{4P'_{\mathrm{Ca}}}{P_{\mathrm{Cs}}}[\mathrm{Ca}^{2+}]_{\mathrm{o}}}{\dfrac{P_{\mathrm{Na}}}{P_{\mathrm{Cs}}}[\mathrm{Na}^{+}]_{\mathrm{i}} + \dfrac{P_{\mathrm{K}}}{P_{\mathrm{Cs}}}[\mathrm{K}^{+}]_{\mathrm{i}} + \dfrac{4P'_{\mathrm{Ca}}}{P_{\mathrm{Cs}}}[\mathrm{Ca}^{2+}]_{\mathrm{i}}} \right) \tag{121}$$

$$P'_{\mathrm{Ca}} = \frac{P_{\mathrm{Ca}}}{1 + \exp\left(\dfrac{VF}{RT}\right)} \tag{122}$$

Sodium potassium pump current – I_{NaK}

$$I_{\mathrm{NaK}} = I_{\mathrm{NaK}}\ f_{NaK}\ k_{NaK}\ n_{NaK} \tag{123}$$

$$f_{NaK} = \frac{1}{\left(\begin{array}{c} 1 + 0.125\exp\left(\dfrac{-0.1VF}{RT}\right) + \\ 0.00219\exp\left(\dfrac{[\mathrm{Na}^+]_\mathrm{o}}{49.71}\right)\exp\left(\dfrac{-1.9VF}{RT}\right) \end{array}\right)} \tag{124}$$

$$k_{NaK} = \frac{1}{1 + \left(\dfrac{K_{m,\mathrm{K}}}{[\mathrm{K}^+]_\mathrm{o}}\right)^{\mathrm{n_K}}} \tag{125}$$

$$n_{NaK} = \frac{1}{1 + \left(\dfrac{K_{m,\mathrm{Na}}}{[\mathrm{Na}^+]_\mathrm{i}}\right)^{\mathrm{n_{Na}}}} \tag{126}$$

Sodium calcium exchanger current – I_{NaCa}

$$I_{\mathrm{NaCa}} = \frac{\bar{I}_{\mathrm{NaCa}}\ f_{allo}\left([\mathrm{Na}^+]_\mathrm{i}^3[\mathrm{Ca}^{2+}]_\mathrm{o} f_{2,NaCa} - [\mathrm{Na}^+]_\mathrm{o}^3[\mathrm{Ca}^{2+}]_\mathrm{i} f_{1,NaCa}\right)}{(1+k_{sat}f_{1,NaCa})\left(\begin{array}{c} K_{m,\mathrm{Cao}}[\mathrm{Na}^+]_\mathrm{i}^3 + K_{m,\mathrm{Nao}}^3[\mathrm{Ca}^{2+}]_\mathrm{i} + \\ [\mathrm{Ca}^{2+}]_\mathrm{o}[\mathrm{Na}^+]_\mathrm{i}^3 + [\mathrm{Na}^+]_\mathrm{o}^3[\mathrm{Ca}^{2+}]_\mathrm{i} + \\ K_{m,\mathrm{Nai}}^3[\mathrm{Ca}^{2+}]_\mathrm{o}\left(1+\frac{[\mathrm{Ca}^{2+}]_\mathrm{i}}{K_{m,\mathrm{Cai}}}\right) + \\ [\mathrm{Na}^+]_\mathrm{o}^3 K_{m,\mathrm{Cai}}\left(1+\left(\frac{[\mathrm{Na}^+]_\mathrm{i}}{K_{m,\mathrm{Nai}}}\right)^3\right) \end{array}\right)} \quad (127)$$

$$f_{1,NaCa} = \exp\left((\gamma-1)\frac{VF}{RT}\right) \quad (128)$$

$$f_{2,NaCa} = \exp\left(\gamma\frac{VF}{RT}\right) \quad (129)$$

$$f_{allo} = \frac{1}{1+\left(\frac{K_{m,\mathrm{Allo}}}{[\mathrm{Ca}^{2+}]_\mathrm{i}}\right)^{\mathrm{n_{Allo}}}} \quad (130)$$

Membrane potential - V

$$\frac{dV}{dt} = -\begin{pmatrix} I_{\mathrm{CaL}} + I_{\mathrm{CaT}} + I_{\mathrm{Na}} + I_{\mathrm{h}} + I_{\mathrm{Cl(Ca)}} + I_{\mathrm{NSCC}} + I_{\mathrm{NaK}} + I_{\mathrm{NaCa}} + \\ I_{\mathrm{KCNQ1}} + I_{\mathrm{KCNQ4}} + I_{\mathrm{KCNQ5}} + I_{\mathrm{hERG}} + I_{\mathrm{K1}} + I_{\mathrm{K2}} + I_{\mathrm{Ka}} + I_{\mathrm{K(Ca)}} \end{pmatrix} \quad (131)$$

$[\mathrm{Ca}^{2+}]_{\mathrm{i}}$ **dynamics**

$$\frac{d[\mathrm{Ca}^{2+}]_{\mathrm{i}}}{dt} = -(J_{\mathrm{Ca,mem}} + J_{\mathrm{NaCa}} + J_{\mathrm{PMCA}}) \quad (132)$$

Ca^{2+} **flux from membrane calcium channels** – $J_{\mathrm{Ca,mem}}$

$$J_{\mathrm{Ca,mem}} = \beta \frac{C_m A_c}{z_{Ca} F V_c} (I_{\mathrm{CaL}} + I_{\mathrm{CaT}} + I_{\mathrm{NSCC,Ca}}) \quad (133)$$

Ca^{2+} **flux from sodium calcium exchanger** – J_{NaCa}

$$J_{\mathrm{NaCa}} = \beta \frac{C_m A_c}{z_{Ca} F V_c} I_{\mathrm{NaCa}} \quad (134)$$

Ca^{2+} **flux from plasma membrane** Ca^{2+}**-ATPase** – J_{PMCA}

$$J_{\mathrm{PMCA}} = \frac{\bar{J}_{\mathrm{PMCA}}}{1 + \left(\frac{K_{m,\mathrm{PMCA}}}{[\mathrm{Ca}^{2+}]_{\mathrm{i}}} \right)^{\mathrm{n_{PMCA}}}} \quad (135)$$

$[\mathrm{Ca}^{2+}]_{\mathrm{i}}$**-dependent force**

$$\mathrm{Force} = \bar{F}\ \omega \quad (136)$$

$$\omega_\infty = \frac{1}{1 + \left(\frac{K_{m,\mathrm{F}}}{[\mathrm{Ca}^{2+}]_{\mathrm{i}}} \right)^{\mathrm{n_F}}} \quad (137)$$

$$\tau_\omega = 4000 \left(0.235 + \frac{1 - 0.235}{1 + \left(\frac{[\mathrm{Ca}^{2+}]_{\mathrm{i}}}{K_{m,\mathrm{F}}} \right)^{\mathrm{n_F}}} \right) \quad (138)$$

$$\frac{d\omega}{dt} = \frac{\omega_\infty - \omega}{\tau_\omega} \quad (139)$$
